# Genetic Diversity among *Enterococcus faecalis*

**DOI:** 10.1371/journal.pone.0000582

**Published:** 2007-07-04

**Authors:** Shonna M. McBride, Vincent A. Fischetti, Donald J. LeBlanc, Robert C. Moellering, Michael S. Gilmore

**Affiliations:** 1 Schepens Eye Research Institute, Harvard Medical School, Boston, Massachusetts, United States of America; 2 The Laboratory of Bacterial Pathogenesis and Immunology, The Rockefeller University, New York, New York, United States of America; 3 Antibacterial Molecular Sciences, Global Research and Development, Pfizer, Inc., Ann Arbor, Michigan, United States of America; 4 Department of Medicine, Beth Israel Deaconess Medical Center, and Harvard Medical School, Boston, Massachusetts, United States of America; University of Toronto, Canada

## Abstract

*Enterococcus faecalis,* a ubiquitous member of mammalian gastrointestinal flora, is a leading cause of nosocomial infections and a growing public health concern. The enterococci responsible for these infections are often resistant to multiple antibiotics and have become notorious for their ability to acquire and disseminate antibiotic resistances. In the current study, we examined genetic relationships among 106 strains of *E. faecalis* isolated over the past 100 years, including strains identified for their diversity and used historically for serotyping, strains that have been adapted for laboratory use, and isolates from previously described *E. faecalis* infection outbreaks. This collection also includes isolates first characterized as having novel plasmids, virulence traits, antibiotic resistances, and pathogenicity island (PAI) components. We evaluated variation in factors contributing to pathogenicity, including toxin production, antibiotic resistance, polymorphism in the capsule (*cps*) operon, pathogenicity island (PAI) gene content, and other accessory factors. This information was correlated with multi-locus sequence typing (MLST) data, which was used to define genetic lineages. Our findings show that virulence and antibiotic resistance traits can be found within many diverse lineages of *E. faecalis.* However, lineages have emerged that have caused infection outbreaks globally, in which several new antibiotic resistances have entered the species, and in which virulence traits have converged. Comparing genomic hybridization profiles, using a microarray, of strains identified by MLST as spanning the diversity of the species, allowed us to identify the core *E. faecalis* genome as consisting of an estimated 2057 unique genes.

## Introduction

Enterococcal species are core constituents of the intestinal flora of many animal species ranging from humans to flies [1]. Enterococci have gained notoriety over the past few decades as frequent causes of multiple antibiotic resistant, hospital-acquired bloodstream, urinary tract and surgical wound infections; and because of their capacity to transfer antibiotic resistances to other microbes [2]–[5]. Although more than a dozen different enterococcal species have been associated with human disease, the majority of human enterococcal infections are due to the species *Enterococcus faecalis* [4]–[8].

The ability of *E. faecalis* isolates to cause serious infections has been linked to the intrinsic ruggedness of the bacterium, which allows the organism to persist in the hospital environment and survive many host defenses, compounded by the acquisition of a variety of variable virulence traits by horizontal transfer from other organisms [9]–[12]. Examples of variable traits that are known or suspected of enhancing the virulence of the organism, include the cytolysin toxin, a gelatinase, enterococcal surface protein Esp, aggregation substance, a hyaluronidase, and a bile salt hydrolase [4], [7], [10], [13]–[16]. Enterococci employ mechanisms, such as pheromone-induced plasmid exchange, and contact dependent plasmid and transposon exchange, to disseminate these traits [4].

Treatment of *E. faecalis* infections is often confounded by antibiotic resistance. Beyond the comparatively high level of resistance that is intrinsic to the species [17], acquired genes confer resistance to chloramphenicol, clindamycin, erythromycin, tetracycline, high-level aminoglycosides, beta-lactamase, and vancomycin [7]. *E. faecalis* strain V583 represented the first vancomycin-resistant enterococcal isolate in the U.S. [18]. Its genome sequence consisted of more than 25% mobile or foreign DNA elements [19]. In 2002 a transposon well documented in enterococci, was discovered in a vancomycin-resistant clinical isolate of *Staphylococcus aureus* [12], [20], strongly implicating *E. faecalis* in the dissemination of resistances to other species of clinical importance.

Recently, multi-locus sequence typing (MLST) schemes have been developed to facilitate analysis of a number of bacterial species [21], including *E. faecalis* [22]–[24]. The development of a facile means for characterizing the genetic background of a strain makes possible the study of the flow of mobile elements within the species. Previous studies using MLST to differentiate isolates of *E. faecalis* at a subspecies level centered on identifying virulent clusters and outbreak isolates, as well as plotting the emergence of antibiotic resistance elements in the population [22]–[24]. In this study, we utilize MLST to define the diversity of the *E. faecalis* species and to determine the core genome content.

A collection of 21 *E. faecalis* strains were previously assembled by Maekawa and coworkers [25] from approximately 1,000 isolates and from pre-existing collections, to represent the diversity of the *E. faecalis* species as judged by serotyping. Since the genetic basis for serological identity was unknown, it was of interest to determine the extent to which serologic diversity reflected genetic diversity, and to determine the genetic relationships among these strains. The diversity of this collection was expanded by examining additionally 85 isolates from outbreaks, clinical strains of special interest (*i.e.* by the discovery of novel traits), commensal strains, animal isolates, and strains collected from the pre-antibiotic era. To determine the extent to which traits associated with virulence and antibiotic resistance had penetrated into the species, these strains were examined for elements of the *E faecalis* pathogenicity island [10], antibiotic resistance, capsule locus polymorphism, and other traits associated with *E. faecalis* strains of increased pathogenic potential. To determine the approximate size and composition of the core *E. faecalis* genome, and to comprehensively assess the penetrance of variable traits identified within the genome of vancomycin resistant strain V583 into the rest of the species lineages, strains representing the deepest nodes spanning the unrooted cladogram derived from MLST data, were compared by microarray.

## Materials and Methods

### Bacterial strains and culture methods

*E. faecalis* strains used in this study are listed in Table 1. These isolates were selected for analysis based on the following criteria: prior use in *E. faecalis* diversity studies (*i.e.*, development of serotyping methods for strain identification), diverse dates of isolation, association with disease, occurrence in healthy flora, adoption for use in the laboratory, and historic significance–*i.e*., isolates from the pre-antibiotic era, association with the discovery of novel virulence determinants, or other notable factors. The isolation date listed for each strain is based on specific isolation source data when available, or the earliest known publication in which a strain is mentioned.

**Table 1:** Bacterial strains used in this study

Strain	Isolation date	Source	MLST	Synonyms and Description	References
T1	≤1950	unknown	**21**	SS498; CDC reference strain; from Y. Ike	25, 40, 116, 117
T2	≤1992	urine	**11**	Sapporo-603; Sapporo, Japan; from Y. Ike	25, 40, 116, 117
T3	≤1992	urine	**67**	Sapporo-109; Sapporo, Japan; from Y. Ike	25, 40, 116, 117
T4	≤1992	urine	**62**	Otaru-104; Otaru, Japan; from Y. Ike	25, 40, 116, 117
T5	≤1992	urine	**68**	Kobe-16148; Kobe, Japan; from Y. Ike	25, 40, 116, 117
T6	≤1992	urine	**63**	Tokyo-74; Tokyo, Japan; from Y. Ike	25, 40, 116, 117
T7	≤1992	urine	**64**	Nagasaki-213; Nagasaki, Japan; from Y. Ike	25, 40, 116, 117
T8	≤1992	urine	**8**	Nagasaki-742; Nagasaki, Japan; from Y. Ike	25, 40, 116, 117
T9	≤1992	urine	**69**	Tokyo-10; Tokyo, Japan; from Y. Ike	25, 40, 116, 117
T10	≤1992	urine	**70**	Osaka-34; Osaka, Japan; from Y. Ike	25, 40, 116, 117
T11	≤1992	urine	**65**	Sapporo-027; Sapporo, Japan; from Y. Ike	25, 40, 116, 117
T12	≤1992	urine	**36**	Okinawa-C1; Okinawa, Japan; from Y. Ike	25, 40, 116, 117
T13	≤1992	urine	**21**	Sapporo-6144; Sapporo, Japan; from Y. Ike	25, 40, 116, 117
T14	≤1992	urine	**9**	Tokyo-91; Tokyo, Japan; from Y. Ike	25, 40, 116, 117
T15	1973	wound	**40**	1824-73; U.S.; from Y. Ike	25, 40, 116, 117
T16	≤1951	infant/fecal	**19**	NCTC8729, s161 type 3; isolated from infant in U.K.; from Y. Ike	25, 40, 76, 116, 117
T17	≤1951	infant/fecal	**66**	NCTC8734, B8 type 8; isolated from infant in U.K.; from Y. Ike	25, 40, 76, 116, 117
T18	≤1951	infant/fecal	**71**	NCTC 8730, GB122 type 4; isolated from infant in U.K.; from Y. Ike	25, 40, 76, 116, 117
T19	≤1951	infant/fecal	**91**	NCTC 8744, D36 type 19; isolated from infant in U.K.; from Y. Ike	25, 40, 76, 116, 117
T20	≤1951	infant/fecal	**22**	NCTC 8745, N161 type 20; isolated from infant in U.K.; from Y. Ike	25, 40, 76, 116, 117
T21	≤1951	infant/fecal	**30**	NCTC 8731, N83 type 5; isolated from infant in U.K.; from Y. Ike	25, 40, 76, 116, 117
F1	early 1900s	milk	**72**	ATCC 376, L36[4]; isolated by S. Orla-Jensen	
SS-7	1918	cheese	**72**	Lancefield C1; from R. Facklam	115
ATCC 4200	3/23/1926	blood	**105**	R.F.1; rheumatic fever isolate	119, 120
SS-6	1930s	unknown	**21**	Lancefield D76; from R. Facklam	
X98	1934	infant/fecal	**19**	Lancefield H69D6, ATCC 27276	39, 121, 138
ATCC 6055	≤1937	milk	**113**	In1, NCTC5957	
D173	7/16/1939	blood	**112**	18085 (R. Lancefield via V. Fischetti)	
ATCC 19433	≤1942	ref strain	**25**	NCTC775, DSM20478, JCM8726, NCDO581, Tissier strain; control strain for Group D	128, 130
ATCC 10100	≤1948	ref strain	**114**	P-60, NCIB7432, NCIB8644; originally from R. Williams as *L. mesenteroides*. Used in assay of riboflavin.	134
RMC1	2/18/1954	clinical	**90**	54×40; from the collection of Roger M. Cole of the NIAID via D. LeBlanc	
RMC5	12/14/1954	clinical	**53**	54×518; from the collection of Roger M. Cole of the NIAID via D. LeBlanc	
B653	4/25/1956	blood/endo	**111**	10D; blood culture of endocarditis patient (R. Lancefield via V. Fischetti)	
E1	1960s	endocarditis	**40**	MGH Boston, MA; U.S.; from R. Moellering	142, 143
RM3817	1960s	blood	**40**	3817; MGH Boston, MA, U.S.; from R. Moellering	98
RM4679	1960s	blood	**9**	4679; MGH Boston, MA, U.S.; from R. Moellering	98
E1Sol	1960s	fecal	**93**	stool surveillance sample from antibiotic-naive population, Solomon Islands	144
Ned10	1961	horse	**9**	D5278/61; Netherlands; from R. Willems	
ATCC 27275	≤1962	unknown	**40**	X52 (from P.H. Koppen)	138
RMC65	11/21/1963	unknown	**110**	63×35; from the collection of Roger M. Cole of the NIAID via D. LeBlanc	
39-5	≤1964	oral	**94**	oral isolate from periodontitis (from Rosan&Williams); contains at least 6 known plasmids; from D. Clewell	75, 139, 145
FA2-2	≤1973	clinical	**8**	U.K.; Rif/Fus resistant mutant derived from plasmid-free strain JH2 (Jacob and Hobbs); common laboratory strain	33, 136
JH1	≤1974	clinical	**40**	isolated in U.K.; Kan/Strep/Erm/Tet resistant isolate containing multiple plasmids; from D. Clewell; common laboratory strain	33, 137, 141
DS5	≤1974	unknown	**55**	FDA strain PCI1326, ATCC 14508, NCDO2131; Erm/Tet resistant isolate containing plasmids α, β, and γ; from D. Clewell	11, 140
ATCC 29200	≤1974	urogenital	**21**	8413; Quebec, Canada; bacteriophage host	135
OG1RF	≤1975	oral	**1**	ATCC 47077; plasmid-free, Rif/Fus resistant mutant of OG1; common laboratory strain	42, 43
ATCC 27959	≤1975	cow	**40**	NADC A-12; bovine mastitis isolate, Iowa, U.S.	129
5952	≤1976	clinical	**30**	Ann Arbor, MI, U.S.; contains plasmids pOB1? from D. Clewell	72, 75
DS16	≤1978	clinical	**40**	Ann Arbor, MI, U.S.; contains plasmids pAD1? from D. Clewell; Tet/Erm/Strep/Kan resistant	9, 74, 136, 146
RC73	≤1979	clinical	**40**	Ann Arbor, MI, U.S.; contains 5 known plasmids; Tet resistant; from D. Clewell	75
ATCC 35038	1980s	chicken	**59**	NCTC 11428, F87/268, PB21; intestine of young chicken	147, 148
HH22	≤1982	urine	**6**	Houston, TX, U.S.; Erm/Tet/Amp/Gent resistant isolate; first identified β-lactamase producing *E. faecalis;* from B. Murray	22, 24, 41, 149
A-2-1	early 1980s	infant/sepsis	**62**	Denver, CO, U.S.; outbreak of neonatal sepsis (1980-1984), from R. Facklam	127
A-3-1	early 1980s	infant/sepsis	**40**	Denver, CO, U.S.; outbreak of neonatal sepsis (1980-1984), from R. Facklam	127
B-4-111	early 1980s	infant/sepsis	**95**	Denver, CO, U.S.; outbreak of neonatal sepsis (1980-1984); from R. Facklam	127
SF19	mid 1980s	clinical	**6**	Michigan, U.S.; Gent resistant isolate; from M. Zervos	
MMH594	1985	blood	**6**	Wisconsin, U.S.; Erm/Gent resistant; first identified and sequenced pathogenicity island; common laboratory strain	10, 126
SF100	mid 1980s	clinical	**6**	California, U.S. Gent/Strep resistant; from M. Zervos	150
SF105	mid 1980s	clinical	**9**	California, U.S. Gent/Strep resistant; from M. Zervos	150
SF339	1986	clinical	**106**	Virginia, U.S.; Gent resistant; contains Tn924; from M. Zervos	131, 132
SF350	1986	clinical	**64**	Winnipeg, Canada; Gent resistant; contains Tn924 and multiple plasmids; from M. Zervos	131, 132
SF370	1986	clinical	**6**	Cleveland, OH, U.S.; Gent resistant; contains Tn924; from M. Zervos	131, 132
WH571	Nov-86	urine	**9**	Connecticut, U.S.; Gent/Pen/Cm/Erm/Tet/Kan/ Strep resistant, β-lactamase-producing isolate; from J. Patterson	122-124, 133
CH19	Jul-87	wound	**9**	Boston, MA; Gent/Pen/Erm/Tet/Strep/Kan resistant, β-lactamase-producing isolate; from L.B. Rice	118
WH245	≤1987	urine	**9**	West Haven, Connecticut, U.S.; Cm/Strep/Erm/Tet/Pen resistant, β-lactamase-producing isolate; from J. Patterson	122, 123, 125, 133
WH257	≤1987	urine	**9**	West Haven, Connecticut, U.S.; Gent/Strep/Erm/Tet/Pen resistant, β-lactamase-producing isolate; from J. Patterson	122, 123, 125, 133
CH570	≤1987	blood	**6**	Canonsburg, PA, U.S.; Gent/Cm/Amp resistant, β−lactamase-producing isolate; from J. Patterson	122-124, 133
V583	2/12/1987	blood	**6**	ATCC700802; St. Louis, MO, U.S.; First isolated Vancomycin-resistant and first sequenced *E. faecalis* genome	18, 19
V587	2/26/1987	urine	**6**	St. Louis, MO, U.S.; Van resistant; (different patient from V583)	18
CH116	1987-1988	fecal	**9**	Boston, MA; U.S.; Gent/Kan/Strep/Tet/Erm/Pen resistant, β−lactamase-producing isolate; from L.B. Rice	118
CH136	1987-1988	urine	**9**	Boston, MA; U.S.; Gent/Kan/Strep/Tet/Erm/Pen resistant, β−lactamase-producing isolate; from L.B. Rice	118
CH188	late 80s	liver	**9**	Boston, MA; U.S.; Gent/Kan/Strep/Tet/Erm/Cm?/Pen resistant, β-lactamase-producing isolate; from L.B. Rice	118
SF1592	late 80s	clinical	**6**	Delaware, U.S.; β-lactamase-producing isolate; from M. Zervos	
SF5039	1/1/1991	urine	**64**	Michigan, U.S.; Van resistant isolate; from M. Zervos	
SF6375	10/1/1991	clinical	**64**	Michigan, U.S.; Van resistant isolate; from M. Zervos	
YI6-1	≤1992	clinical	**28**	Japan; Tet resistant, plasmid-free derivative of YI6; first isolate characterized with chromosomal-encoded cytolysin; from Y. Ike	31
TR161	10/23/1993	blood	**6**	Buffalo, NY, U.S. (Sisters Hospital); from T. Russo	
TR197	10/30/1993	blood	**109**	Buffalo, NY, U.S. (Buffalo Gen. Hosp.); from T. Russo	
599951	3/6/1994	blood	**64**	Chicago, IL, U.S.; Van resistant; from M. Hayden	
SF21520	mid 1990s	blood	**6**	Valencia, Spain; Van resistant; from M. Zervos	151
SF21521	mid 1990s	blood	**28**	Valencia, Spain; Van resistant; from M. Zervos	151
12030	mid 1990s	clinical	**64**	Cleveland, OH, U.S.	83, 152, 153
12107	mid 1990s	clinical	**21**	Cleveland, OH, U.S.	83, 152, 153
79-3	10/4/1999	blood	**64**	Chicago, IL, U.S.; Van resistant; from M. Hayden	
AR01/DG	8/1/2001	dog*	**108**	New Zealand; dog wound isolate; First isolated bacitracin resistant isolate; Van/Erm/Tet resistant; *same as common Van resistant chicken isolates in N.Z.; from J. Manson	154
SF24396	2001	urine	**21**	Michigan, U.S.; from M. Zervos	
SF24397	2001	urine	**2**	Michigan, U.S.; from M. Zervos	
SF24413	2002	urine	**2**	Michigan, U.S.; Van resistant isolate; from M. Zervos	155
SF26630	2002	urine	**6**	Michigan, U.S.; Van resistant isolate; from M. Zervos	155
HIP11704	2002	clinical	**4**	Michigan, U.S.; Van/Erm strain co-isolated from VRSA patient (VanA); from L. Weigel	142
Merz89	7/6/2002	blood	**40**	89; Johns Hopkins Hosp., Maryland, U.S.; *esp*+ isolate; from W.G. Merz	156
Merz96	5/3/2002	blood	**103**	96; Johns Hopkins Hosp., Maryland, U.S.; Van resistant isolate; from W.G. Merz	156
Merz151	12/6/2002	blood	**104**	151; Johns Hopkins Hosp., Maryland, U.S.; Van resistant isolate; from W.G. Merz	156
Merz192	5/5/2002	blood	**40**	192; Johns Hopkins Hosp., Maryland, U.S.; esp+ isolate; from W.G. Merz	156
Merz204	7/11/2002	blood	**40**	204; Johns Hopkins Hosp., Maryland, U.S.; *esp*+ isolate; from W.G. Merz	156
SF28073	2003	urine	**2**	Michigan, U.S.; Van resistant isolate; from M. Zervos	155
Pan7	3/5/2005	commensal	**21**	Panose 7; fecal sample of healthy volunteer; Boston, MA, U.S.	
Fly1	7/5/2005	drosophila	**101**	commensal isolate of wild-captured fly; Oklahoma, U.S.; isolated by C. Cox	
Fly 2	2005	drosophila	**102**	commensal isolate of Oregon R Bloomington fly stock (immediately upon arrival); isolated by C. Cox	
Com1	2/1/2006	commensal	**34**	fecal sample of healthy volunteer; Boston, MA, U.S.	
Com2	2/1/2006	commensal	**34**	fecal sample of healthy volunteer; Boston, MA, U.S.	
Com6	2/1/2006	commensal	**21**	fecal sample of healthy volunteer; Boston, MA, U.S.	
Com7	2/1/2006	commensal	**107**	fecal sample of healthy volunteer; Boston, MA, U.S.	
D1	unknown	pig	**40**	73-30082-2; Denmark; from L.B. Jensen	
D3	unknown	pig	**47**	73-30245-2; Denmark; from L.B. Jensen	
D6	unknown	pig	**16**	73-30318-4; Denmark; from L.B. Jensen	

*E. faecalis* strains were routinely grown on Difco brain heart infusion (BHI) agar (1.5% w/v, Difco), or in BHI without aeration, at 37 °C. Genomic DNA was isolated as described [26], and PCR was performed using standard protocols [27]. All strains used in this study were verified as being of the species *faecalis* by PCR, using the species-specific ddl-1 and ddl-2 primers [28].

### MLST analysis

Sequencing of alleles for MLST was performed by the DNA Sequencing Center for Vision Research (DSCVR) at Massachusetts Ear and Eye Infirmary, using an Applied Biosystems BigDye Terminator V3.1 Cycle Sequencing Kit. Sequencing reactions were resolved using an ABI Prism 3100 genetic analyzer. A standard set of *E. faecalis* MLST primers were used for amplification and sequencing as described (http://efaecalis.mlst.net; [21]) and are listed in Table S1. The seven genes evaluated for MLST of *E. faecalis* are *aroE, gdh, gki, gyd, pstS, xpt,* and *yqiL*. For each isolate, each gene was amplified a minimum of two times and sequenced with the specific forward or reverse primer a minimum of three times. Sequence types of isolates are defined by the allelic profile at these seven loci, with each unique combination of alleles assigned a distinct sequence type number. Once an allelic profile for each isolate was established, dendrograms were created by an unweighted pair-group method with arithmetic averages, using the ‘View Tree’ link after Batch Query analysis, as previously described [24]. Isolates with the same allelic profile, and therefore the same sequence type, are regarded as members of a single clone or lineage. Clonal complexes were defined as groups of isolates that differed in no more than two of the seven loci analyzed and consisted of single and double-locus variants of a founder isolate determined using eBURST v3 (data not shown; http://www.mlst.net, [24]).

### Dot blot hybridization

Total DNA from enterococcal strains was isolated from overnight cultures grown in BHI, and 500 ng was spotted onto Hybond-N^+^ nylon membranes (Amersham). DNA was fixed by UV crosslinking with 70,000 µJ/cm^2^. Membranes were washed in 2×SSC buffer [27] and blotted dry. Hybridization was carried out using the DIG-High Prime DNA Labeling and Detection Starter Kit I (Roche Diagnostics), per manufacturer's instructions. PCR products used for probes included amplified internal fragments of a putative bile acid hydrolase, *cbh;* capsule locus *cpsF;* cytolysin locus *cylB;* biofilm-related protein encoding *esp;* gelatinase, *gelE;* putative stress regulator, *gls-24-*like (EF0117); putative glycosyl hydrolase (EF0077); putative nuclease (EF0031); *S. pneumoniae psaA* Mn transporter homolog (EF0095); bifunctional aminoglycoside inactivating gene *aac6′-aph2″;* and the chloramphenicol acetyltransferase gene, *cat*. Each was amplified using primers listed Table S1. To sample genomes for the presence of portions of the *E. faecalis* pathogenicity island (PAI), genes from across the pathogenicity island were selected as shown in Fig S1; sampled regions of the PAI did not include the 5′ most region that contained high homology to plasmid pAM373, due to the extrachromosomal and highly variable nature of this DNA in isolates. Genes *blaZ* and *ermB* were detected by PCR using primer pairs ermB-1/ermB-2 and blaZ-1/blaZ-2, respectively. β-lactamase activity was confirmed by colorimetric assay. Due to the large diversity of tetracycline resistance determinants, genotyping for tetracycline resistance was performed only by PCR for the most common resistance determinants, *tetL*, and *tetM* [29].

### Cytolysin transmissibility

The cytolysin operon has been shown to occur on highly transmissible plasmids, such as pAD1 [30], and within the chromosome [31], where it has been identified to be encoded within a pathogenicity island [10]. Portions of the pathogenicity island, including the cytolysin, transfer at a very low rate [10], [32]. To obtain evidence as to whether the cytolysin operon occurred on a highly transmissible plasmid in strains found to be positive for this trait, candidate donor strains, and a recipient strain, *E. faecalis* FA2-2 (which possesses chromosomal markers for rifampicin and fusidic acid resistance [33]), were independently grown overnight at 37°C in BHI. Cultures were then diluted 1:10, combined in equal proportions, and 50 ul spotted onto non-selective BHI agar plates and incubated overnight. The resulting mixed colony was then streaked onto BHI agar containing 5% horse blood 50 µg/ml rifampicin and 25 µg/ml fusidic acid, to select for isolated recipient colonies. Streptomycin and spectinomycin (both at 500 µg/ml, using resistant strain JH2SS as the recipient) were substituted for selection of transconjugants of strain YI6-1 due to inherent rifampicin and fusidic acid resistance. Hemolytic colonies were checked for the presence of the unselected resistance, rifampicin, to verify their status as transconjugants. Conjugation tests were performed in duplicate.

### Capsule Locus Polymorphism

Maekawa serotyping strains T1, T5, and T2 were previously shown to harbor prototype capsule locus polymorphisms [34]. The *cps* locus of Maekawa strain T1 consists of *cpsA,* and *B*, followed by the non-capsule related *hcp1* gene [34]. The *cps* locus of Maekawa strain T2 consists of *cpsA, B, C, D, E, F, G, H, I, J, K,* followed by *hcp1*. The *cps* locus of Maekawa strain T5 consists of *cpsA, B, C, D, E, G, H, I, J, K,* followed by *hcp1*, with *cpsF* conspicuously absent [34].

PCR tests were designed to distinguish CPS T1, T2 and T5 type polymorphisms. Primer pair cpsB5-F/hcp1-R (Table S1) was designed to generate an amplification product of 950 bp from CPS T1 type strains, as the primers are complementary to *cpsB* and *hcp1* (EF_2484). Primers cpsEend-F/5′cpsG-R were designed to amplify the region between *cpsE* and *cpsG*, to detect the presence of *cpsF,* which distinguishes CPS T2 and T5 polymorhpisms. An amplification product of 1098 bp indicated the presence of *cpsF* characteristic of the T2 capsule type. Generation of a product of 199 bp indicated the presence of *cpsE* and *cpsG*, but the absence of cpsF as is characteristic of the CPS T5 polymorphism. All of the strains examined yielded CPS locus PCR products consistent with one of the three known polymorphisms.

### Comparative genomic hybridization

#### Microarray Chip Design

A custom Affymetrix GeneChip, SLARE1 (St. Louis Antibiotic Resistant *Enterococcus*-1), was designed to contain a total of 3582 probe sets to: 1) the 3182 predicted ORFs from the chromosome of strain V583 (GenBank AE016830) [19]; 2) additional pathogenicity island genes of strain MMH594 known not to be represented in V583 (GenBank AF454824) [10]; and 3) *E. faecalis* plasmid and antibiotic resistance genes or clusters from other *E. faecalis* strains for which nucleotide sequences had been reported: *vanA* operon (GenBank AY697425), *blaZ* (GenBank M60253), *bcr* operon (GenBank AY496968), *vanG* operon (GenBank DQ212986), *vanE* operon (AF430807), *tetM* (GenBank X56353), pRE25 plasmid/*cat* (X92945), pTEF1 (AE016833), pTEF2 (AE016831), and pTEF3 (AE016832). Microarrays included additionally 111 Affymetrix designed eukaryotic and prokaryotic negative control probe sets.

Each probe set consisted of 14 perfect-match/single base-mismatch oligonucleotides per predicted ORF. A total of 42 ORFs from strain V583 were not represented on the array due to a combination of synthesis constraints on the potential probes and cross-hybridization between them and hard prune (repetitive) elements. A full listing of probe sets including genes represented in and excluded from the microarray and experimental data are available in the accompanying supplementary materials deposited in the ArrayExpress public repository at http://www.ebi.ac.uk/arrayexpress under accession number E-MEXP-1090. Details of the algorithm used in construction of custom Affymetrix GeneChips are available at the manufacturer's website: www.affymetrix.com/technology/index.affx.

#### Bacterial DNA isolation

Based on their representation of the deepest phylogenetic nodes within the MLST dendrogram, genomic DNA of strains ARO1/DG, Com6, Fly1, HIP11704, D6, and JH1 was isolated from individual duplicate cultures as described above. DNA from strains MMH594 and V583 were included as positive controls. All post-DNA isolation comparative genomic microarray protocols were performed by Genome Explorations, Inc., Memphis, TN. Prior to fragmentation and labeling, the purity and concentration of genomic DNA samples were determined from A_260/280_ readings using a dual beam UV spectrophotometer. Genomic DNA integrity was determined by capillary electrophoresis using the DNA 12000 Lab-on-a-Chip kit and the Bioanalyzer 2100 (Agilent Technologies), per manufacturer's instructions. The extent of DNA fragmentation produced by DNase I treatment (see below) was determined by capillary electrophoresis using the DNA 1000 Lab-on-a-Chip kit and Bioanalyzer 2100 (Agilent Technologies), per manufacturer's instructions.

#### DNA fragmentation and labeling

DNA samples were adjusted to 0.25N NaOH (Sigma), heated to 65°C and purified using QIAquick columns (Qiagen) to remove contaminating RNA. 4 µg of DNA was fragmented with DNase I (0.6 U/μg, Promega), denatured at 95°C, and then labeled with biotinylated dd-UTP using the Bioarray™ Terminal Labeling Kit (Enzo). Briefly, each sample of fragmented DNA was incubated with 20 µl of 5× Reaction Buffer, 10 µl of 10× CoCl_2_, 1 µl of 100× Biotin-ddUTP, and 2 µl of 50× Terminal Deoxynucleotide Transferase for 2 hr at 37°C. Reactions were terminated by addition of EDTA (pH8.0) to a final concentration of 6 mM.

#### Oligonucleotide array hybridization and analysis

Labeled genomic DNA fragments were adjusted to contain 0.06M MES-Na buffer (Sigma), 2.7 M TMACl (Sigma), 5% DMSO (Sigma), 0.01% Tween-20 (Sigma), 2.5× Denhardt's solution, and 0.1 mg/ml herring sperm DNA (Promega), and hybridized for 16 hr at 48°C to the custom designed *E. faecalis* genome array SLARE1 (Affymetrix). Arrays were washed at 25°C with 6 × SSPE (0.9 M NaCl, 60 mM NaH_2_PO_4_, 6 mM EDTA)+0.01% Tween-20 followed by a stringent wash at 50°C with 0.6× SSPE+0.01% Tween-20. The arrays were then stained with phycoerythrein-conjugated streptavidin (Molecular Probes) and the fluorescence intensities were determined using the GCS 3000 high-resolution confocal laser scanner (Affymetrix). The scanned images were analyzed using programs resident in GeneChip Operating System v1.4 (GCOS; Affymetrix). GCOS-generated signal intensity values and detection calls for probe sets covering prokaryotic and eukaryotic control sequences, and *E. faecalis* sequences, were used to assess hybridization quality and specificity after standardization of each array. Standardization was accomplished by global scaling the average of the fluorescent intensities of all probe sets on an array to a constant target intensity of 250 for all arrays used. Scale factors produced by global scaling were similar to normalization factors generated using 50% trimmed mean signal intensity values.

#### Comparative genotyping and bioinformatic analyses

To identify absent or divergent sequences in each strain (relative to the published sequences for the V583 genome, the *E. faecalis* pathogenicity island, plasmids pTEF1, pTEF2, pTEF3 and pRE25, the *vanE, vanG* and *bcr* operons, and the *vanA, tetM*, and *blaZ* genes), GCOS-generated detection calls for each probe set were first converted to numerical values (A = 0 [absent], M = 0.5 [ambiguous], P = 1.0 [present]) and averaged from duplicate experiments for each strain. Average signal intensity values were calculated for probe sets with detection values = 1.0 in all eight strains (overall AvgSig_Present_ = 332.86). By comparison, the average signal intensity value for probe sets with detection values = 0 in all eight strains was 29.85. A final detection call of Present (or non-divergent) was assigned to each probe set in a strain if two criteria were met: 1) log_2_ [strain average signal value/average signal value for all 8 strains] ≥−1.5, and 2) log2 [strain average signal value/Overall AvgSig_Present_] ≥−3.0. These criteria set a limit on strain to strain variability and an absolute signal intensity requirement for a call of Present (or non-divergent). Relative strain divergence was assessed based on 1) the percentage of non-divergent probe sets called “present” in each strain and 2) Pearson correlation coefficients. Dendrograms depicting relative strain divergences were generated in GeneMaths XT (Applied Maths) based on a similarity matrix of Pearson correlation coefficients using the Combined Linkage algorithm. Cluster maps aligned strains according to similarity dendrogram and probe sets according to 1) the number of strains in which each probe set was called Present, and then by COG designation, or 2) the genomic order specified in the GCOS-generated CHP file. Cluster maps were generated in GeneMaths XT. Gene descriptions and COG designations were obtained from the National Center for Biotechnology Information (NCBI).

Microarray values were ambiguous for the two-component histidine kinase and response regulator HK14 and RR14 (EF_1209 and EF_1210) for strains Fly1 and Aro1/DG, and for the histidine kinase HK17 (EF_1632) for strains MMH594 and V583. These loci were confirmed present by PCR using primer sets detailed in Table S1.

### Phenotype Assays

#### β-lactamase assay

Direct assessment of β-lactamase activity was performed using the colorimetric Nitrocefin disk assay (Remel Co., U.K.), per manufacturer's instructions.

#### Cytolysin assay

Blood agar plates were used for the qualitative detection of hemolytic activity. Plates contained Bacto brain-heart infusion and 1.5% agar, to which PBS washed horse erythrocytes were added to a final concentration of 5% (v/v). Strains were streaked for isolation on blood agar and assessed after 24 hours for zones of hemolysis surrounding colonies [35].

#### Bile salt hydrolase (CBH) assay

Isolates were grown overnight at 37°C in brain-heart infusion broth, and 5 µl were spotted onto plates containing 70 g/l Difco MRS Lactobacilli medium, 0.37 g/l calcium chloride (Sigma-Aldrich), and 0.5% taurodeoxycholic acid (Sigma-Aldrich #T0557), hereafter referred to as CBH agar [36]. *E. faecalis* strains were grown for 48 hours and examined for precipitation of deconjugated taurodeoxycholic acid.

#### Gelatinase assay

Gelatinase expression was detected by the observance of a halo around isolated colonies streaked on BHI skim milk agar [37]. Strains for which no halo of clearing around colonies was observed following 48 h of incubation at 37 °C were considered phenotypically negative.

#### Antibiotic resistance assays

All strains were tested for antibiotic resistance using a single-concentration assay, analogous to that developed for detection of high-level aminoglycoside resistance [38]. The following antibiotics and concentrations were used to assess resistance: gentamicin, 500 µg/ml; erythromycin, 50 µg/ml; chloramphenicol, 10 µg/ml; tetracycline, 10 µg/ml; ampicillin, 4 µg/ml and 8 µg/ml; and vancomycin, 4 µg/ml and 8 µg/ml (Sigma-Aldrich). Assays were performed with fresh cells (approximately 10^6^ CFU) from BHI agar suspended in 100 µl BHI medium containing antibiotic. Growth was monitored after 24 hours (48 hours for vancomycin resistance), and compared to known positive and negative controls. Experiments were performed a minimum of two times with analogous results.

#### Chloramphenicol acetyltransferase assay

Isolates identified as resistant to chloramphenicol by microdilution assay, but probe negative for the *cat* gene by PCR and dot blot, were assayed specifically for CAT activity using a *FAST CAT*® Green (deoxy) chloramphenicol acetyltransferase detection kit, and analyzed by thin-layer chromatography as recommended by the manufacturer (Molecular Probes, U.S.A.).

## Results

### Phylogenetic analysis of *E. faecalis* serotyping isolates

**Figure 1:**
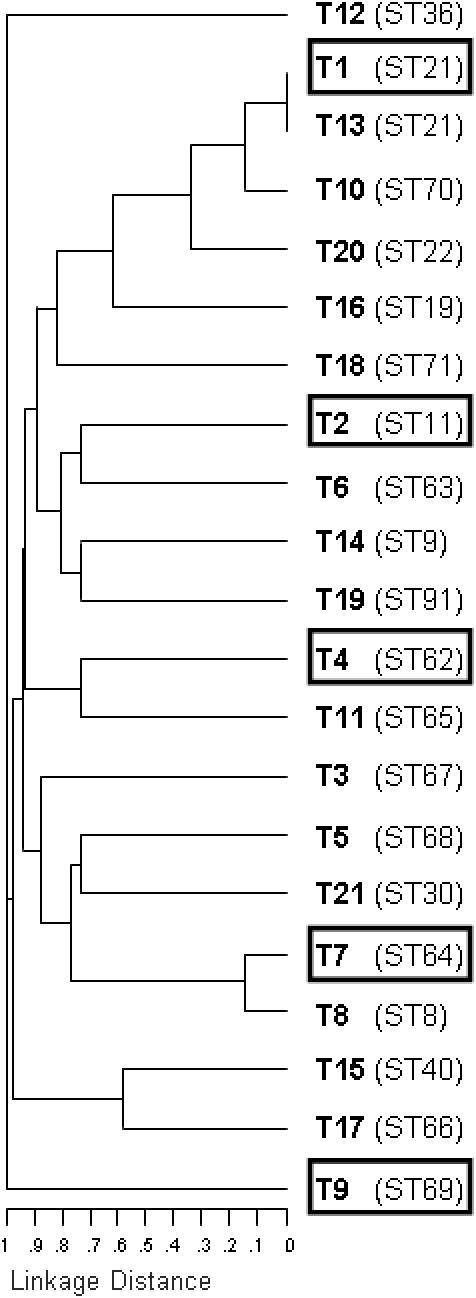
Dendrogram created from serological typing strains using the *E. faecalis* MLST database efaecalis.mlst.net. Multi-locus sequence typing (MLST) of *E. faecalis* isolates was based on sequences of internal gene fragments for 7 housekeeping genes. Each gene variation (for each of the seven genes) is assigned a unique allele number. The combination of the 7 allele numbers (allelic profile) for each strain defines the multi-locus sequence type, or ST. The relatedness of isolates based on sequence type is shown as an unrooted cladogram, determined by (UPGMA) analysis of the allelic profiles. Boxed isolates represent the most common serotypes found in human populations in previous studies [25], [40].

In 1992, S. Maekawa and her colleagues [25] developed a refined serotyping test for the rapid identification of the majority of *E. faecalis* strains. Rabbit antisera were exhaustively cross adsorbed against a battery of *E. faecalis* isolates, including a smaller serotype diverse set previously created by M.E. Sharpe in 1964 [39]. Upon examining 832 strains from the U.S., Japan, and the U.K. in their study, over 78% of all isolates fell into one of 21 proposed serotypes, with serotypes 1, 2, 4, 7, and 9 representing the majority of isolates [25], [40]. Because of the established diversity of this set of *E. faecalis* serotyping strains, it represented an ideal starting point for determining the genetic diversity of *E. faecalis* strains. Using MLST, we assessed the genetic relatedness of the 21 Maekawa type strains (T1-T21). A dendrogram depicting the relationships between these strains is shown in Fig. 1. These results indicate that the 21 serotypes of *E. faecalis* in fact span a great amount of species diversity and identify genetically distinct lineages. Only 2 of the 21 strains, strain T1 and T13, were found to be of the same sequence type.

### Test for additional species diversity

**Figure 2:**
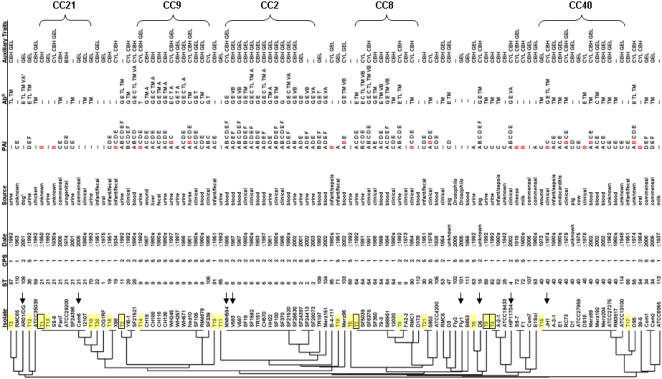
Dendogram of isolates aligned with capsule type, pathogenicity island segments, and antibiotic resistance traits. MLST-based dendrogram showing genetic relationship of all *E.* faecalis isolates in this study. Small yellow highlights indicate a serotyping type strain, while black boxes designate the five most common serotypes [40]. Arrows designate isolates used for comparative genomic microarray analysis. Abbreviations are defined as follows: ST = sequence type; CPS = capsule type; PAI = pathogenicity island fragment outlined as letter designations in Fig. S1 (A = *nuc1*; B = *cylB*; C = *esp*; D = hydrolase homolog similar to *xylS*; E = *psaA* homolog; F = *gls-24* like); A red letter B indicates strains that readily transfer cytolysin via mating; Ab^R^ = antibiotic resistance; T = tetracycline resistance; TM = *tetM*+; TL = *tetL*+; E = *ermB*+; VA = *vanA*+; VB = *vanB*+; G = gentamicin resistant; C = *cat+*; A = *blaZ*+; CBH = bile salt hydrolase; GEL = gelatinase; CYL = cytolysin. More detailed strain information is listed in Table 1.

The extent to which the Maekawa serotyping strains represented the diversity of the *E. faecalis* species, was tested by examining additionally 85 strains drawn from diverse ecologies and points in time. This set includes strains isolated from New Zealand to Boston, and dates ranging from the early 1900s to 2006. The collection also included clinical specimens, hospital-unrelated fecal isolates, and strains from non human sources ranging from Drosophila to swine. MLST analysis was performed on these additional isolates, listed in Table 1, and the genetic relationship of these strains to the Maekawa strains is shown in Figure 2.

Within this diverse origin strain set, some sequence types (hereafter STs) occurred multiple times. The five most common STs were ST40 (n = 13), ST6 (n = 12), ST9 (n = 11), ST21 (n = 8), and ST64 (n = 7), which include 2 of the 5 serotypes identified as common by serotyping [40]. Additionally, these five most common STs had closely related single locus variants (SLVs) and/or double locus variants (DLVs) in our collection. The largest clonal clusters (CCs) in this diversity collection, defined here as a ST represented by three or more isolates, along with any SLVs and DLVs of these sequence types, include: CC21 (consisting of ST21 and ST70), CC9 (ST9 and ST106), CC2 (ST6 and ST2), CC8 (ST64, ST8, ST90, and ST112), and CC40 (ST40 and ST114). In all, the five most common clonal groups encompassed 58% of the 106 isolates examined by MLST in this study.

Interestingly, of the *E. faecalis* strains that have been used for most laboratory studies, namely strains V583 (first *E. faecalis* genome sequenced) [19], MMH594 (first pathogenicity island description) [10], HH22 (first β-lactamase positive isolate) [41], FA2-2 [33], and OG1RF [42], [43], only one common sequence type is represented: ST6 (HH22, V583, and MMH594). We previously showed extensive sequence conservation within the 129 genes of the pathogenicity island and the 11 genes of the capsule locus of V583 and MMH594 [10], and others have observed that these strains and HH22 are closely related in genes that define the MLST assessment loci [22]. In this study, as in [24], these strains were identified as ST6. OG1RF was identified as representing the rarer sequence type ST 1, and FA2-2 is ST8.

When the MLST data of the Maekawa *et al*. [25] serotyping strains T1-T21 (Fig. 1) are viewed on the dendrogram in Fig. 2, these isolates (highlighted in yellow) in fact span the diversity of sequence types at many grades of relatedness. This was somewhat surprising given that the molecular basis for interaction of agglutinating serotype anti-serum with the surface of the each of the sequence types is unknown, except for type 2 [44].

Of the isolates in our collection from the era preceding the widespread use of antibiotics (which we defined as having been acquired ≤1951), strains are found throughout the dendrogram, with only one isolate out of fifteen (SS-6) belonging to one of the most common STs of this study (ST21). No obvious clustering of older isolates is observed.

### Capsule locus polymorphisms and distribution among isolates

All strains from Table 1 were analyzed for genes associated with virulence in *E. faecalis,* or homologs linked to virulence in other organisms (see Table 2 and Fig. 2). Isolates were first examined at the capsule locus (*cps*) to determine which of the known capsule polymorphisms occurred. The *cps* capsule locus of *E. faecalis* was discovered in part using Maekawa serotyping antiserum (serotype 2/strain T2). The T2 cps operon consists of 11 open reading frames, designated *cpsA* through *cpsK* [34], [44], [45]. Based on observed *cps* operon polymorphisms [34], primers were designed to assess these polymorphisms within our collection. Maekawa ‘type’ strains T1-T21 were verified as possessing the combinations of the 11 known genes of the *cps* operon previously inferred from restriction fragment length polymorphism and dot blot analysis (35, 77), and to verify the basis for the 3 previously identified polymorphisms (35, 77) using primers listed in Table S1. For all of the Maekawa serotype strains tested, the three known capsule operon polymorphisms were found: 1) that which includes all 11 genes as in strain T2 (designated CPS type 2); 2) that which includes all genes except for *cpsF* as in Maekawa strain T5 (CPS type 5); or only *cpsA* and *cpsB* as found in Maekawa strain T1 (CPS type 1).

**Table 2:** Bacterial virulence determinants and putative virulence factors examined.

Bacterial Determinant	Accession no.	Putative function	Reference	Method of detection
PAI				
* nuc-1*	EF0031	nuclease (homolog)	10	PCR, Southern hybridization
* cylA,B,&M*	EF0046-48	cytolysin production	35	PCR, Southern hybridization for *cylB*
* esp*	EF0056	enterococcal surface protein	10	PCR, Southern hybridization
hydrolase	EF0077	glycosyl hydrolase (*xylS* homolog)	10	PCR, Southern hybridization
* psaA*	EF0095	metal binding protein (homolog)	157	PCR, Southern hybridization
* gls24*-like	EF0117	general stress protein	10, 158	PCR, Southern hybridization
* cbh*	EF0040	bile salt hydrolase	10	PCR, Southern hybridization, CBH assay
Other				
* cps*	EF0085-95	capsular polysaccharide	44	PCR, Southern hybridization
* gelE*	D85393	gelatinase	68, 159, 160	PCR, Southern hybridization, Gelatinase assay
* fsrB*	EF1821	accessory gene regulator	68	PCR
Antibiotic resistance				
* vanA*	X56895	D-Ala-D-Lac ligase / Vancomycin resistance	52, 54	PCR, microdilution assay
* vanB*	L06138	D-Ala-D-Lac ligase / Vancomycin resistance	53, 54	PCR, microdilution assay
* ermB*	U86375	adenine methylase/ erythromycin resistance	55	PCR, microdilution assay
* cat*	X92945	chloramphenicol acetyl-transferase/ chloramphenicol	51	PCR, Southern hybridization, resistance assay *FAST CAT* assay
* tetM*	X92947	ribosomal protection / tetracycline resistance	48	PCR, Southern hybridization, resistance assay
* tetL*	NC_005013	efflux pump / tetracycline resistance	47	PCR, Southern hybridization, resistance assay
* aac6'-aph2"*	M13771	bifunctional enzyme / high-level aminoglycoside resistance	50	PCR, Southern hybridization, resistance assay
* blaZ*	M60253	β-lactamase / β-lactam resistance	49	PCR, nitrocefin assay, resistance assay

A limited primer set, optimized to detect the differences between these 3 capsule operon polymorphisms, was then used to determine which of the three CPS types were present in the remaining isolates of the collection. All strains tested yielded one of the three CPS polymorphisms, based on characteristic PCR products. Dot blots were performed on the entire collection, using as a probe an internal fragment of *cpsF,* to confirm presence or absence of the *cpsF* gene, which is characteristic of CPS2, and to verify that negative PCR results did not derive from point changes within primer hybridization sites. The occurrence of CPS 1, 2 and 5 polymorphisms among the strains studied, as identified by this approach, is shown in Figure 2. Wherever the collection contained multiple isolates within a sequence type, CPS type was invariant among those strains. The most common CPS type among the diversity of lineages was type 1. When strains were examined by decade of isolation, CPS type 1 remained most common.

### Antibiotic resistances and determinants among isolates

Single-concentration broth assays were performed to test the resistance of isolates to antibiotics from six different classes: gentamicin, erythromycin, ampicillin, vancomycin, tetracycline, and chloramphenicol. For many isolates, some antibiotic resistance information was available. Reanalysis confirmed those results, with the exception of strain CH19, for which erythromycin resistance was not detected by phenotype. This strain was, however, found to harbor the *ermB* resistance gene. Based on the broth assay results, 58% of isolates exhibited resistance to tetracycline, 38% to erythromycin, 33% to high-level gentamicin, 14% to vancomycin, 11% to chloramphenicol, and 9% to ampicillin. An additional 6 strains exhibited low-level (≤4 µg/mL) ampicillin resistance, but were β-lactamase negative (strains F1, RMC1, SF5039, SF21520, SF21521 and Merz96). Tetracycline resistance was the only antibiotic resistance trait that occurred among isolates included in this study known to be of commensal origin [46]. Of isolates tested, 31% (34/106) were sensitive to all six antibiotics. All strains isolated prior to 1954 were susceptible to all of the antibiotics tested. Less than half of CC21 isolates, which includes sequence types 21 and 70, had any antibiotic resistance, with tetracycline being the sole resistance found. Strains of CC40, which includes sequence types 40 and 114, also exhibited relatively few antibiotic resistance factors, with most isolates having only one type of resistance.

**Figure 3:**
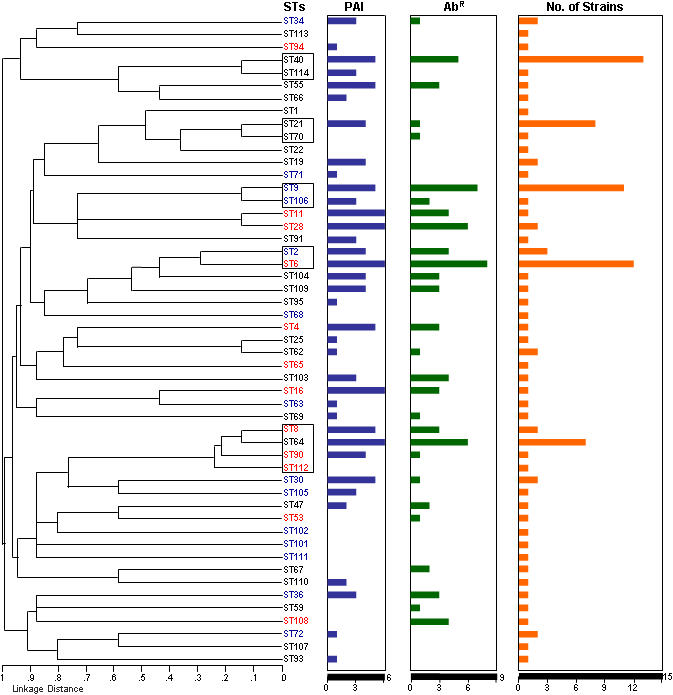
Dendrogram and composite virulence determinants among sequence types. MLST-based dendrogram compiling data from isolates of the 51 identified sequence type lineages. PAI = number pathogenicity island genes present per lineage; Ab^R^ = number of antibiotic resistance determinants per lineage. Brackets encompass abundant clonal isolates and their single and double locus variants where applicable.

Clonal clusters CC9, CC2, and CC8 contained strains that harbored many resistances, with some isolates carrying determinants for resistance to five antibiotic classes. Vancomycin resistance, conferred by the *vanA* or *vanB* genetic determinants, was found in 14% of isolates, with most occurring in CC2 and CC8 (10/15). Ampicillin resistance conferred by β-lactamase was only found in isolates from CC9 and CC2 of our collection, specifically in sequence types 9 and 6 (Fig. 2 and Table 3), and was the rarest among this collection (Table 3). The number of antibiotic resistances found in each genetic lineage is illustrated in Fig. 3.

**Table 3:** Distribution of putative virulence genes in five most common clonal groups.

clonal group	CC21	CC9	CC2	CC8	CC40	non-clustering	Total from groups
**# of isolates**	9	12	15	11	14	45	61
***bla***	**0** (0/9)	**58** (7/12)	**20** (3/15)	**0** (0/11)	**0** (0/14)	**0** (0/45)	**16** (10/61)
***cat***	**0** (0/9)	**50** (6/12)	**20** (3/15)	**18** (2/11)	**7** (1/14)	**2** (1/45)	**20** (12/61)
***vanA/B***	**0** (0/9)	**0** (0/12)	**40** (6/15)	**36** (4/11)	**0** (0/14)	**11** (5/45)	**16** (10/61)
***ermB***	**0** (0/9)	**67** (8/12)	**87** (13/15)	**64** (7/11)	**14** (2/14)	**24** (11/45)	**49** (30/61)
***aac6'-aph2"***	**0** (0/9)	**67** (8/12)	**100** (15/15)	**27** (3/11)	**0** (0/14)	**16** (7/45)	**43** (26/61)
***tetM/L***	**44** (4/9)	**58** (7/12)	**40** (6/15)	**82** (9/11)	**79** (11/14)	**40** (18/45)	**61** (37/61)
***gls-24*** **-like**	**0** (0/9)	**0** (0/12)	**67** (10/15)	**9** (1/11)	**0** (0/14)	**18** (8/45)	**18** (11/61)
***cylB***	**22** (2/9)	**33** (4/12)	**47** (7/15)	**36** (4/11)	**29** (4/14)	**31** (14/45)	**34** (21/61)
***nuc1*** *	**0** (0/9)	**83** (10/12)	**100** (15/15)	**55** (6/11)	**43** (6/14)	**31** (14/45)	**61** (37/61)
***hyd*** *	**11** (1/9)	**75** (9/12)	**93** (14/15)	**73** (8/11)	**7** (1/14)	**38** (17/45)	**54** (33/61)
***esp***	**33** (3/9)	**92** (11/12)	**27** (4/15)	**73** (8/11)	**93** (13/14)	**40** (18/45)	**64** (39/61)
***psaA*** *	**33** (3/9)	**75** (9/12)	**93** (14/15)	**82** (9/11)	**64** (9/14)	**42** (19/45)	**72** (44/61)
***cbh***	**44** (4/9)	**100** (12/12)	**80** (12/15)	**100** (11/11)	**93** (13/14)	**56** (25/45)	**85** (52/61)
***fsr***	**67** (6/9)	**100** (12/12)	**100** (15/15)	**0** (0/11)	**100** (14/14)	**67** (30/45)	**77** (47/61)
***gelE***	**100** (9/9)	**100** (12/12)	**100** (15/15)	**100** (11/11)	**100** (14/14)	**96** (43/45)	**100** (61/61)

Groups are comprised of abundant clonal isolates (n>3) and their single and double locus variants as determined by MLST analysis (Fig 3) and eBURST [24]: CC21 = ST21&70, CC9 = ST9&106, CC2 = ST2&6, CC8 = ST8, 64, 90, &112, and CC40 = ST 40&114. Numbers in bold indicate percentage of isolates positive for the specified genotype for a given grouping as calculated from the number of positive versus total isolates in parentheses. Genes investigated above the dashed line encode antibiotic resistance determinants, while those below the dash are known to the pathogenicity island of *E. faecalis* and/or code for auxiliary enzymes (see Table 2). Antibiotic resistance genotypes encoded by genes other than those listed are not included in this table. * = putative nuclease and glycosyl hydrolase genes

**Figure 4:**
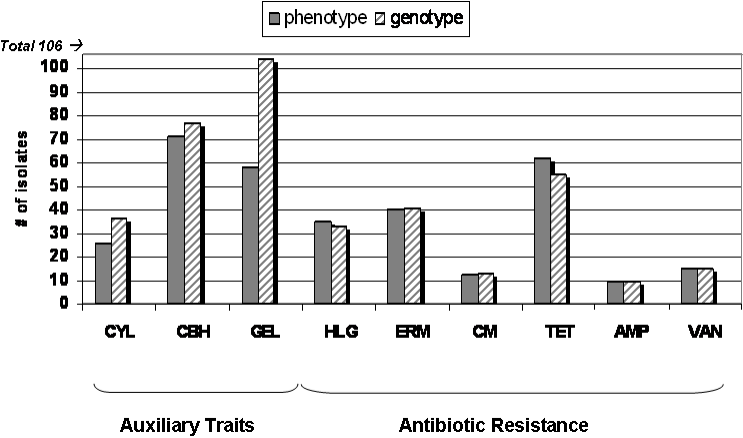
Virulence-associated phenotypes and corresponding genotype for all isolates. Phenotypes were determined by microdilution assay for antibiotic resistance or enzyme-specific tests for auxiliary enzymatic traits. Genotypes were determined by PCR amplification and/or hybridization for genes known to encode each phenotype. A positive genotype is indicated by the presence of one or more genes known to produce a given phenotype (e.g. genotypically positive vancomycin strains may contain the *vanA* or *vanB* genes—a strain containing both would only be counted once).

Strains were tested by PCR and dot blot for the antibiotic resistance genes most commonly conferring these resistances in *E. faecalis*. These included *tetM* and *tetL* for tetracycline resistance [47], [48], *blaZ* for β-lactamase-mediated ampicillin resistance [49], *aac6′-aph2″* for aminoglycosides [50], *cat* for chloramphenicol [51], *vanA* and *vanB* for glycopeptides [52]–[54], and *ermB* for macrolide resistance [55] (Table 2 and Fig. 2). Comparing antibiotic resistance phenotype and genotype, we found that all erythromycin resistant strains were positive for *ermB* (Fig. 4). Further, all vancomycin resistant isolates contained either *vanA* or *vanB* ligases, and all high-level ampicillin resistant (>4 µg/mL) isolates expressed β-lactamase activity and possessed the gene, *blaZ*. The majority of tetracycline resistant isolates (54 of 62) carried *tetM*, one isolate was positive for *tetL*, and 12 of the 54 *tetM* positive isolates also carried the *tetL* gene. The remaining 7 tetracycline-resistant strains tested negative for either *tetM* or *tetL* by PCR, and may be resistant via one of the rarer tetracycline resistance mechanisms not examined [29]. Most gentamicin resistant isolates (32/34) were positive by PCR and dot blot for the *aac6′-aph2″* bifunctional enzyme that confers aminoglycoside resistance [50], with the exceptions of strain JH1 and YI6-1, which had high-level gentamicin resistance and were not positive for *aac6′-aph2″.* JH1 was previously described as having a 3′5″-aminoglycoside phosphotransferase type III resistance gene [56]. The aminoglycoside resistance mechanism for YI6-1 was not further explored.

Chloramphenicol resistance was associated with the presence of the chloramphenicol acetyl-transferase gene, *cat,* by PCR and dot blot, for all chloramphenicol resistant strains except RM4679, WH245, and WH571, which were phenotypically chloramphenicol resistant, but were negative for the *cat* gene by these tests. Because chloramphenicol acetyl transferase (CAT) is the most common mechanism for chloramphenicol resistance in enterococci [57], [58], we further evaluated CAT activity in these isolates using the *FAST CAT* chloramphenicol acetyltransferase kit. CAT activity was confirmed for all three strains, suggesting that they harbor a divergent *cat* gene [57]. The number of isolates having the tested antibiotic resistance phenotypes and genotypes listed above are illustrated in Fig. 2.

### Known and suspected virulence traits

A number of auxiliary traits have been identified that participate in colonization or virulence in *E. faecalis,* or are similar to those of known activity in other intestinal pathogens [59], [60]. We examined our collection of isolates for assayable activities associated with virulence, and corresponding coding sequences: cytolysin, gelatinase, and bile salt hydrolase (Fig 2). Cytolysin and gelatinase production were characterized in *E. faecalis* over 40 years ago [61]–[63], and many studies have linked these variable traits of the species to enterococcal virulence [64], [65]. Bile salt hydrolase is expressed by some strains of *E. faecalis,* and also by intestinal pathogens [66], [67]. It is known to contribute to a bacterium's ability to survive in the gastrointestinal tract [15]. Analysis of phenotypes and corresponding genotypes for cytolysin, gelatinase, and bile salt hydrolase activity, revealed more isolates that were genotypically positive than phenotypically positive in laboratory tests (Fig. 4). The greatest difference between genotype and detectable phenotype was for gelatinase activity (58 gelatinase positive of 104 *gelE* ^+^), then cytolysin (26 cytolysin positive of 36 *cylA/B/M* ^+^), followed by bile salt hydrolase (71 hydrolase positive of 77 *cbh* ^+^). For gelatinase, the lack of phenotype in the presence of a positive genotype in many cases has been attributed to the absence of a known *gelE* regulator *fsrB* [68]. In the present study, 27 of the 46 *gelE* positive isolates that did not display a gelatinase positive phenotype were *fsrB* negative by PCR, while all gelatinase positive isolates tested positive for *fsrB* (Table S2). All three auxiliary trait activities assayed were present in the major MLST groups and branches of the cladogram lineages, with the exception of gelatinase activity, which was not present in any isolate of CC8. Upon further examination, it was found that all of the isolates of CC8 tested positive for the gelatinase gene, *gelE*, but negative for the *fsrB* regulator of gelatinase expression [68].

### Comparative genomic hybridization analysis of diverse *E. faecalis* isolates, and classification of core genetic elements

**Figure 5:**
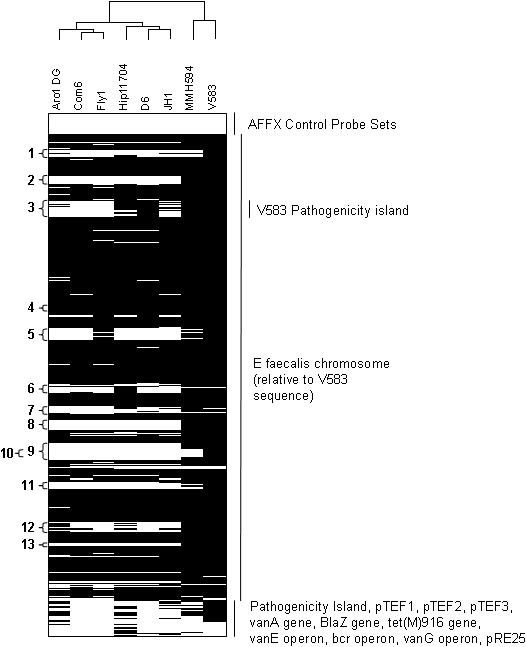
Visualization of final absent and present calls for all probe sets across 8 distinct *E. faecalis* isolates. Comparative genomic hybridization was performed on DNA from isolates V583, MMH594, JH1, HIP11704, D6, ARO1/DG, Com6, and Fly1 as described in materials and methods. Probe sets are ordered according to the *E. faecalis* V583 gene sequence and the Affymetrix library file. Absent probe sets are in white; present probe sets are in black. Clustering of the strains was based on complete linkage using the Pearson correlation coefficient for the Absent/Present calls (A = 0, P = 1). The same clustering pattern is generated when average signal intensity values are used instead of Absent/Present calls (not shown). The numbered regions on the left correspond to the following previously identified and unidentified mobile genetic regions from strain V583: 1) EF_0125-EF_0166, 2) EF_0302-EF_0355 (PHAGE01), 3) EF_0479-EF_0628 (V583 PAI), 4) EF_1275-EF_1293 (PHAGE02), 5) EF_1416-EF_1489 (PHAGE03), 6) EF_1847-EF_1897, 7) EF_1987-EF_2043 (PHAGE04), 8) EF_2084-EF_2145 (PHAGE05), 9) EF_2240-2282/EF_2335-2351, 10) EF_2284-EF_2334 (Tn/*vanB*), 11) EF_2512-EF_2545, 12) EF_2798-EF_2856 (PHAGE06), and 13) EF_2936-EF_2955 (PHAGE07) (see text).

Based on the MLST relatedness profiles of strains tested, we examined eight strains that spanned the diversity of the species by comparative genomic hybridization. The goal was to identify genes common to maximally diverse strains of *E. faecalis,* thereby defining the core genome. It was further of interest to assess the penetration of variable traits occurring in the V583 genome within diverse lineages of the species. Six of these strains (ARO1/DG, Com6, Fly1, HIP11704, D6, and JH1) represent diverse nodes of the MLST-based cladogram (Fig. 2), unrelated to the known sequence strain, V583. Strain MMH594 was included as an additional positive control to verify detection of genes of the 17 kb portion of pathogenicity island known to be deleted in strain V583 [19]. The results of microarray genome hybridization analysis of the six strains, and V583 and MMH594 controls, demonstrate the relatedness between diverse strains of *E. faecalis* and V583 with respect to its variable gene content (Fig. 5). Probe sets for the array are ordered according to the V583 genome sequence, while additional plasmid and antibiotic resistances genes are arranged independently.

Comparison of divergent *E. faecalis* genomes to strain V583, revealed some V583 genes that were present among all strains tested, and others that were variably present. Variable regions were found to correspond to integrated phages, plasmids, and transposable elements previously identified *in silico* in the V583 genome [19], as well as an additional 60 ORF phage-related variable region not previously identified (region 9). Region 9, present only in V583 and MMH594, is flanked by site-specific recombinases and contains putative metabolic and hypothetical genes, and may represent a new pathogenicity or fitness island. In strain V583, the *vanB* segment (region 10) is flanked by ORFs with high sequence similarity by BLASTp analysis (http://www.ncbi.nlm.nih.gov/blast) to those occuring in mobile elements. Because MMH594 isolation predated that of V583, suggesting that *vanB* was the more recent acquisition, the vancomycin resistance element appears to have inserted into the 60 ORF region 9, which seems to have entered the species in this lineage (ST6). Region 6, a region of the V583 genome previously identified to be atypical in GC composition [19], appears to be extended 8 additional ORFs (EF_1847-EF_1897 instead of EF_1855-EF_1874) based on both the presence of these ORFs in strains identified by comparative genomic analysis, and the presence of a flanking site-specific recombinase. (see microarray data files at http://www.ebi.ac.uk/arrayexpress, accession number E-MEXP-1090). This region is present in the closely related strains V583 and MMH594, and in a divergent strain, D6.

Relatedness based on similarity in gene content was calculated based on the percentage of non-divergent (Present) probe sets in each strain and Pearson correlation coefficients (see supporting microarray information and materials and methods). To the extent that gene content reflects fitness for a particular habitat, this comparison provides a independent approach from MLST for investigating the ecological relationship of *E. faecalis* isolates. As shown in the dendrodram at the top of Figure 5, MMH594 and V583 (both ST6) group together as would be anticipated based on known relatedness in gene content, which includes the pathogenicity island, capsule gene and MLST genes. However, relatedness based on variable gene content did not parallel MLST (Fig. 2), indicating that as expected for mobile elements, variable traits penetrate the species independently of genome sequence drift. Each strain exhibited a minimum identity of 72% with the V583 chromosomal probe sets, and a minimum of 67% of the total *E. faecalis* probe sets (which includes *E. faecalis* genes not found in V583; Table 4). As expected, most of the differences in gene content between the strains tested and V583 appear to stem from variations in putative mobile element content, which is not measurable by MLST. Since only the genome sequence of strain V583 is known, there undoubtedly are many undiscovered variable traits in the non-V583 lineages tested, that if examined could lead to the identification of additional relationships among these strains.

**Table 4:** Present calls for Comparative Genomic Hybridization probe sets by isolate.

Strain	ARO1/DG	Com6	Fly1	JH1	HIP11704	D6	MMH594	V583
Present calls for V583 chromosome	2417	2422	2535	2404	2615	2483	3110	3256
% present calls for V583 chromosome	**73.09**	**73.24**	**76.66**	**72.69**	**79.07**	**75.08**	**94.04**	**98.46**
Present calls in all bacterial probe sets	2493	2425	2566	2407	2739	2559	3210	3409
% present calls in all bacterial probe sets	**69.60**	**67.70**	**71.64**	**67.20**	**76.47**	**71.44**	**89.61**	**95.17**

**Figure 6:**
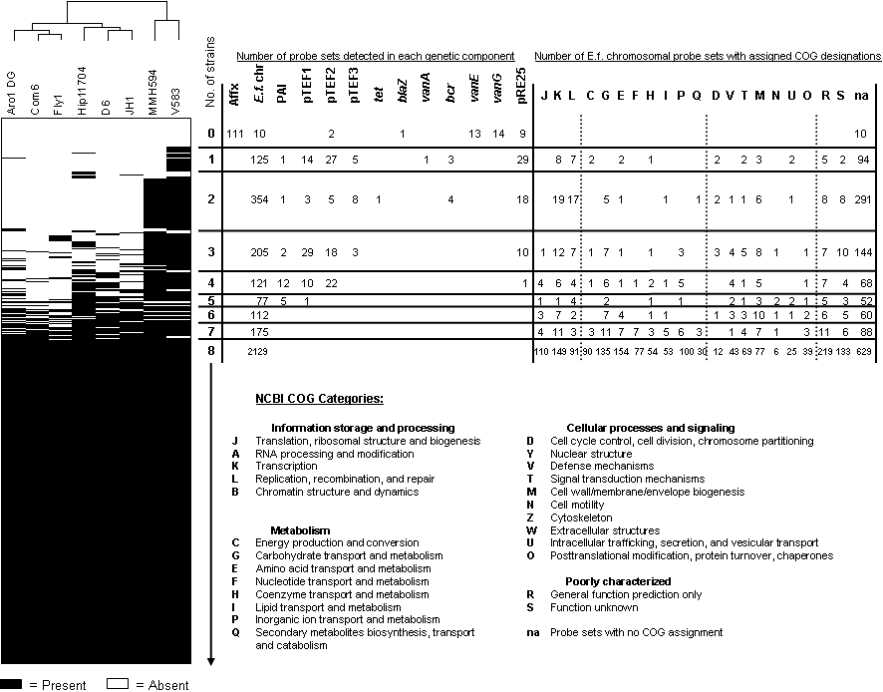
Classification of core and dispensable sequences in 8 strains of *Enterococcus faecalis.* Data from comparative genomic hybridization analysis of isolates V583, MMH594, JH1, HIP11704, D6, ARO1/DG, Com6, and Fly1 are organized by the number of strains for which each probe set is detected (top to bottom). Absent probe sets are in white; present probe sets are in black. Clustering of the strains was based on complete linkage using the Pearson correlation coefficient for the Absent/Present calls (A = 0, P = 1). Genetic elements were classified as part of the core genome if present in all strains tested. COG designations were obtained from the National Center for Biotechnology Information (NCBI). Some genes/probe sets represent more than one COG category.

The core *E. faecalis* genome, as defined by the number of genes present in all 8 strains based on the ORFs included in the microarray, consists of 2057 of the 3091 ORFs (2129 of the 3582 total probe sets, which includes redundancies; Fig. 6). Core and variable genes were categorized by orthologous group, according to NCBI COG designation (Clusters of Orthologous Groups, http://www.ncbi.nlm.nih.gov/COG). Core and accessory genes were further analyzed and ranked by the number of strains for which a present call was made for each probe set, and for predicted cellular role (Fig. 6). All of the core genome ORFs are located on the V583 chromosome; none were associated with extrachromosomal plasmids.

Of probe sets not part of the core genome but common in the majority (five or more) of the eight strains examined, 370 were identified. Of these, 236 probe sets represented poorly characterized or unassigned gene functions, and the remaining 134 spanned all of the general COG categories. Of the seven identified phage regions of the V583 genome, only one is represented in the core genome: PHAGE02 [19]. In addition, the majority (9 of 11) of predicted V583 phosphotransferase systems (PTS) [19] for the transport of sugars are present in the core genome, including those for cellobiose, fructose, glucitol, glucose, maltose, mannitol, mannose, sucrose, and trehalose. Only the galactitol and N-Acetyl galactosamine PTS systems from V583 were not present in all eight isolates. Examination of the core genome for identified two-component signal transduction systems from V583 [69] showed that 12 out of 17 of these systems, as well as an orphaned response regulator, are present in all strains tested. These include HK/RR01, HK/RR02, HK/RR03, HK/RR04, HK/RR05, HK/RR06, HK/RR07, HK/RR09, HK/RR10, HK/RR13, HK/RR14, HK/RR17, and RR18. Furthermore, we found high conservation of genes identified as having a potential role in stress responses [19] within the core genome. Of the 56 stress-response related ORFs in V583, which represent oxidative, osmotic, and metal-ion resistance mechanisms [19], 54 are present in the core genome of *E. faecalis*.

### Strain relatedness and presence of pathogenicity island

A pathogenicity island (PAI) was found to occur in *E. faecalis* strain MMH594, with derivatives in strains V583 and V586 [10]. This region encodes known and putative virulence determinants, surface adhesion factors, carbohydrate metabolism pathways, the cytolysin operon, a putative bile salt hydrolase, as well as many other genes of unknown function and homologs to virulence factors in other bacteria [10]. Many genes in this island, such as the cytolysin operon, have been identified on plasmids, transposons, or other mobile elements, and appear to have integrated into the chromosome at various points in its evolution. In order to survey which regions of the known PAI were present in the strains of our collection, we selected six functionally unrelated genes located across the pathogenicity island to serve as sampling points (Fig. S1). The pathogenicity island (PAI) associated genes we surveyed were present in many nodes of the dendrogram, and in strains from many different sources and dates of isolation. Through PCR and dot blotting for the six PAI genes from Fig. S1, we found that CC9, CC2, and CC8, possessed more of the PAI regions (and also antibiotic resistances) tested than did other clonal clusters (Fig. 3 and Table 3). Bile salt hydrolase, cytolysin, and gelatinase genes, as well as five of six PAI genes sampled (the exception being the *gls24*-like gene) can be found in strains isolated before 1951, including in some of the oldest strains in our collection. The exception, gene EF0117 which specifies a *gls24*-like gene, is located near one end of the PAI and did not appear in strains of our collection until the 1980s in isolates from ST6, of which the majority test positive. The *gls24-like* gene has high homology to the *gls24* gene (EF0080), which has been linked to stress responses in *E. faecalis* [70]. This *gls24*-like gene appears to have penetrated other lineages (STs 11, 28, 36, and 64) in the early 1990's. We found *gls24*-like in isolates of different regions of the dendrogram (Fig. 3), however, it was the rarest of the PAI genes assayed and did not appear in any CC21 or CC40 isolates surveyed.

The nuclease homolog, *nuc1,* (EF0031) exhibits sequence similarity to staphylococcal nucleases that hydrolyze DNA and RNA [71] and is located near the opposite end of the PAI from the *gls24*-like gene (EF0117). *nuc1* was absent in strains of our collection isolated prior to 1950. It was present in every CC2 isolate and was absent from all CC21 isolates, however a pattern of *nuc1* penetration the species is not evident. The other sampled PAI genes, *cylB, esp,* the hydrolase (EF0077), *psaA,* and *cbh,* were found in isolates of our collection dating as far back as 1926; the pattern by which these genes entered the species could not be deduced from this set.

Given that the cytolysin operon is known to be on conjugative plasmids in several of the strains in our collection [11], [43], [72]–[74], we investigated whether the cytolysin determinant of other strains was highly transmissible, as is known for the cytolysin encoding plasmid, pAD1 [75], or on less mobile PAI [10] or other element [31]. Mating experiments showed that 51% (18/35) of the cytolytic strains were able to transmit cytolysin in a mixed colony at a high rate. When aligned to the position of isolates on the dendrogram (Fig. 2), the transmissibility of cytolysin corresponded to specific regions and groups. For instance, all of the cytolysin positive strains in CC21 and CC40 were able to transfer cytolysin in a mixed colony, presumably by conjugation as known for the prototype plasmid pAD1 encoded determinant [75]. Except for strain MMH594, isolates in CC2 that were positive for *cylB* were phenotypically not cytolytic, precluding detection of transconjugants. Transfer of the known PAI-conferred cytolysin determinant within MMH594 was not observed in the mixed colony, consistent with previous observations [32]. Within CC2, V583 and V587 which were isolated from separate patients, are known to carry vestiges of the cytolysin operon within their pathogenicity islands [18]. Part of this operon was deleted in strain V583 and a transposon insertion rendered the V587 cytolysin inactive ([10], V587 data not shown).

Among the eight isolates used for comparative genomic analysis by microarray, no pathogenicity island genes were common to all of the strains, supporting the proposition that it is not part of the core *E. faecalis* genome. Data obtained independently by sampling for the presence of genes within the PAI, for antibiotic resistance, and for other genes detailed in this study were generally supported by the results of the microarray analysis. The sole exception was detection of a previously unknown polymorphism observed in the *cps* locus of strain Fly1, which had no *cpsD* by microarray, but otherwise fit CPS type 5. With respect to genes of the pathogenicity island, the microarray data for the 8 strains tested was consistent with the results of sampling the six PAI loci shown in Fig. S1 (Table 5), validating this set of genes as a useful tool for rapidly probing strains for the presence of various regions of the PAI. Based on the presence of the six representative PAI genes sampled in our collection, we estimate that the middle regions of the PAI between EF0056 (*esp*) and EF0095 (*psaA*) were introduced into *E. faecalis* by the early 1900s, if not before. Comparative hybridization analysis also indicates that the PAI has integrated into multiple MLST lineages, as previously suggested by Nallapareddy *et al.* [22], and is present in strains isolated from both animals and humans (Table 5).

**Table 5:** Presence of 6 representative PAI genes compared to total PAI probe sets by comparative genomic hybridization.

Strain	Com6	Fly1	ARO1/DG	JH1	V583	HIP11704	D6	MMH594
Origin	commensal	insect	dog	clinical	clinical	clinical	pig	clinical
Sequence type (ST)	21	101	108	40	6	4	16	6
6 gene PAI profile^A^	0/6	0/6	0/6	2/6	4/6	5/6	6/6	6/6
CGH PAI probe sets^B^	0/141	0/141	3/141	56/141	125/141	111/141	138/141	137/141

A 6 gene PAI profile includes the nuclease homolog, *cylB, esp, xylS* homolog, *psaA* homolog, and *gls-24* like genes outlined in Fig S1 and Table 2.

B PAI probe sets were designed to the combined V583 and MMH594 PAI sequences (EF_0479-EF_0628 and EF0001-EF0129, respectively) and represent 139 ORFs.

## Discussion

This investigation was undertaken to provide new insights and a global view of the diversity of the species *E. faecalis*. We observed differences between phylogenetically separate groups for pathogenicity island content, antibiotic resistance determinants, and capsule operon polymorphisms, which we hypothesize are due to niche preferences within the species. Variable genetic traits confer niche specialization and virulence to bacteria, including *E. faecalis*. To characterize the diversity of the species, to determine what constitutes the core species characteristics, and to determine the extent of penetration of various traits into the species, we characterized a diverse collection of *E. faecalis* isolates by MLST, comparative genomic hybridization, and directly assessed select genotypic and phenotypic properties. As a starting point, we capitalized on the efforts of previous investigators who, over the past six decades, developed collections of serologically unique *E. faecalis* strains [25], [39], [76]. We complemented these collections with additional isolates from over the past century, and from a variety of geographic locations. Of the 51 different sequence types we identified, the majority (29 sequence types, 57%) had not been detected in previous studies [24], [77].

Our studies show that the previous collections of serologically unique strains were not only antigenically diverse, but remarkably genetically diverse as well. This was somewhat surprising in that expression of a new surface protein, potentially encoded by a mobile element such as a phage or transposon, could generate a novel serotype. Only one example was identified where serological type strains from the Maekawa collection [25] were of the same MLST sequence type–namely T1 and T13, both of which were MLST sequence type 21. The remaining 19 strains of the Maekawa collection constituted 19 sequence types covering most of the unrooted dendrogram that represents the species (Fig 2). The five most common serotypes identified by Maekawa *et al*. [25], [40] in epidemiologic studies, do not appear closely related genetically (serotypes T1, T2, T4, T7, and T9; Figs. 1 and 2; [40]). This suggests that either discrete ecologies select for the presence of these 5 lineages, or that the host mounts a transient immune response promoting a temporal rotation among these lineages. Additional isolates in the collection resolved into 31 additional STs, for a total of 51 STs. Of these 51 STs, 38 were represented only once in our collection. Of the Maekawa serotypes that were reported to be most often isolated in studies of strains from the U.K., U.S.A., and Japan [40], only T1 and T7 were members of MLST clonal clusters deriving from this collection (CC21 and CC8, respectively).

Capsules are typically highly variable traits of pathogens, such as *Streptococcus pneumoniae*, and this variation is driven by evasion of host immune response [78]–[80]. The *cps* capsule locus of *E. faecalis* was discovered in part with the use of Maekawa serotyping antiserum (serotype 2/strain T2), and consists of the 11 open reading frames *cpsA* through *cpsK* [34], [44], [45]. The first vancomycin-resistant isolate, V583, the first characterized pathogenicity island-containing strain MMH594, and a clinical isolate adapted for use in the laboratory FA2-2, were all shown to react with type 2 antiserum, a phenotype that is dependent on the presence of the capsule operon including, in particular, *cpsF* [34], [45].

**Figure 7:**
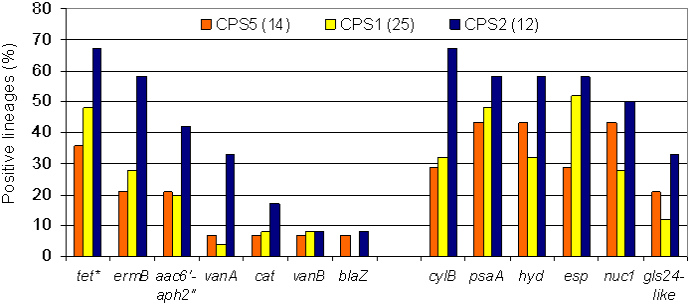
Antibiotic determinants and pathogenicity island composition per sequence type lineage, aligned by capsule locus (*cps*) variation (CPS type). The percentage of sequence type lineages positive for each antibiotic resistance and pathogenicity island listed in Fig 3 were alligned by CPS capsule type and arranged in descending order. Capsule types are invariant for strains within individual sequence type lineages. Details of capsule operon (*cps*) arrangements are outlined in Materials and Methods.

In studies of streptococci, where capsule variation alters serotype, an MLST type was found to correspond to one or more particular serotypes and a given serotype might be common to many clonal complexes or sequence types [81], [82]. Of the 51 sequence types identified, 25/51 were found to be CPS type 1, 14/51 of CPS type 5, and 12/51 of CPS type 2. The vast majority of CPS type 1 lineages harbored 3 or fewer virulence traits (18/25; 72%), or 2 or fewer antibiotic resistances (19/25; 76%) (Fig. 7). Similar proportions were observed for CPS type 5 strains (79% [11/14] possess 3 or fewer virulence traits; 79% [11/14] possess 2 or fewer antibiotic resistance traits). However, half or more CPS type 2 strains (58% [7/12]) harbored 4 or more virulence traits, or 3 or more (58% [7/12]) antibiotic resistance traits (Fig. 7). This observation suggests that CPS2 strains tend to colonize sites where antibiotic resistance genes and virulence traits are advantageous. As the capsule locus, the pathogenicity island, and antibiotic resistances are known not to be physically linked in strain V583 (the only strain for which a genome sequence exists), this observation supports the hypothesis that independent selection has driven the convergence of virulence traits, antibiotic resistances and the type 2 capsule.

Expression of the type 2 capsule has been associated with decreased opsonophagocytosis [44]. That ST6 isolates, which are uniformly of this capsule type and clinical in origin, had the greatest variety of both antibiotic resistances and PAI genes tested, makes the nexus of these traits of special interest in the evolution of highly virulent clones of the species (Fig. 3 and Fig. 7) Throughout our collection, the three known *cps* operon polymorphisms [34], [83] (CPS) were consistent within individual STs, although within an ST variation in antibiotic resistance and virulence traits occurred. This suggests that although the capsule locus polymorphism is a variable trait within the species, it varies at a lower rate than other traits known to reside on mobile elements [84]. Alternatively, it suggests that a change in capsule type may give rise to a new lineage, perhaps by enabling that strain to outcompete the parental type to which the host has responded. That all three CPS variations were found in all of the major branches of the dendrogram supports the hypothesis that there is horizontal transfer. However, a lack of obvious transfer or transposition functions associated with the capsule operon leaves as an open question the mechanism.

The present study revealed variation in the penetration of antibiotic resistances and genes of the pathogenicity island between major clonal clusters, as well as non-clustering isolates. The tetracycline class of antibiotics were discovered in 1945, and put into regular therapeutic use by 1950 [85], [86]. Within 2–3 years, tetracycline resistant strains of enterococci were isolated [86]. The oldest isolate with tetracycline resistance in our collection dated back to 1954. Tetracycline resistance was the only antibiotic resistance trait that occurred among isolates of known commensal origin included in this study. Its occurrence among such strains was known from previous studies of gut flora populations [46]. Tetracycline resistance in enterococci can be the result of genes encoding ribosomal protection mechanisms (TetM-like) or efflux pumps that actively transport antibiotic out of the cell (TetL-like), both of which were found in our strains [87]. Tetracycline and its derivatives are broad-spectrum antibiotics that have been commonly used for the past 67 years to treat bacterial infections, thus their regular use and spectrum of activity has exerted substantial pressure for *E. faecalis* strains to harbor resistance determinants. As such, tetracycline resistance is the most common resistance found in our collection (62/106 isolates) and has penetrated 25 of the 51 sequence types we identified.

Vancomycin, a glycopeptide antibiotic, was discovered in 1952 and was quickly brought to market to combat infections due penicillin resistant bacteria [88]. However, vancomycin use has been limited since its introduction due to problems with toxicity, administration, and the availability of other antibiotics [88]. Resistance of group D streptococci (enterococci) to vancomycin was documented in isolates in 1968 [86], though it is unclear in which specific species this resistance occurred. The use of vancomycin increased during the 1980s, which likely contributed to the rise in resistant organisms that soon followed [88]. Vancomycin resistance in enterococci was encountered in France and the U.K. in 1986, as a result of the *vanA* gene cluster found in enterococcal isolates [89]–[91]. Vancomycin resistance mediated by *vanA* or *vanB* genes was found in only 15/106 strains in our collection. A review of our *vanA*-containing isolates indicates that this genotype first appeared in *E. faecalis* strains in ST6 (CC2) and ST28 (1990s) and was later acquired in ST108, ST104, and ST4. Vancomycin resistance due to the *vanB* genes was found in 1987 in the *E. faecalis* strain V583 [18], which is in ST6 (CC2). *vanB* next appears in ST64 (CC8) in 1991, followed by ST2 and ST103 in 2002. Our study identified vancomycin resistant strains in eight distinct STs, six of which had not been previously identified as harboring *van* genes [24]. The diversity of sequence types harboring *van* genes in our set illustrates an extensive spread of this resistance among *E. faecalis* lineages in less than twenty years since resistance was first discovered.

Ampicillin is a β-lactam antibiotic, the first and most famous of which is penicillin [92]. Discovered in 1928, β-lactams came into widespread use in 1942 as the first class of antibiotics used to treat bacterial infections [93]. In the initial characterization of the bacteriocidal action of penicillin it was clear that the enterococci, including *E. faecalis,* were resistant to the antibiotic [92]. The enterococci have an intrinsic resistance to β-lactam antibiotics that is likely due to a low affinity of the penicillin-binding proteins for the antibiotic [94]. It was not until 1981 that high-level resistance to β-lactams due to penicillinase production was identified in *E. faecalis* in strain HH22 (CC2) [95]. Ampicillin resistance was the least encountered antibiotic resistance in our collection (10/106). The spread of ampicillin resistance conferred by β-lactamase appears to be restricted to two of the most common clonal groups in our set: CC2 and CC8. Penicillin and its derivatives have been heavily used since their inception, thus the lag time between the introduction of β-lactam antibiotics and the discovery of β-lactamase enzymes in *E. faecalis* cannot be attributed to low use. That there is likely little selective advantage for acquiring and maintaining penicillinase enzymes in a intrinsically resistant organism is the most likely reason β-lactamase has not penetrated the species more extensively. Interestingly, the first β-lactamase and vancomycin resistance determinants identified in *E. faecalis* were both independently identified in ST6 strains of CC2.

Chloramphenicol was introduced for therapeutic use in 1949, however its use is currently restricted in the U.S. due to side effects [86]. Resistance among group D enterococci to chloramphenicol was noted immediately upon its inception [96]. In enterococci, this resistance is most commonly associated with the chloramphenicol acetyl-transferase gene, *cat* [57]. We evaluated our isolates for chloramphenicol resistance and found that all 12 chloramphenicol resistant strains were positive for chloramphenicol acetyl transferase. Tracing the introduction of this resistance through our collection, we found that the first isolates with *cat* activity belong to ST9 (CC9), and date back to 1961. Chloramphenicol resistance was then absent from our strains until the mid-1980s, when it occurred in ST9 (CC9) and ST6 (CC2). By 1991, resistance moved to ST64 (CC8), then ST28 in the mid-1990s, and finally ST40 (CC40) in 2002. It is intriguing that of the 12 chloramphenicol resistant isolates we found, only one did not belong to a common clonal cluster (strain SF21521, ST28). Though resistance to chloramphenicol was found in multiple lineages in our collection, the restricted use of chloramphenicol as a therapeutic antibiotic has probably limited the number of resistant organisms in the population by decreasing the selective pressure for maintaining the genetic determinant.

Gentamicin belongs to the aminoglycoside class of therapeutic antibiotics, the first of which (streptomycin) was introduced in 1943 [97]. Enterococci are intrinsically resistant to low levels of aminoglycosides (≤250 µg/ml), while high-level aminoglycoside resistance (≥250–2000 µg/ml) is the result of acquired resistance [98], [99]. High-level acquired resistance can be caused by three different types of aminoglycoside modifying enzymes: phosphotransferases (APHs), acetyltransferases (AACs), or nucleotidyltransferases (ANTs) [100]. The most common source of aminoglycoside resistance in *E. faecalis* involves the bifunctional enzyme *aac6′-aph2″* [50]. We found gentamicin resistance by the *aac6′-aph2″* enzyme most frequently (33/35 gentamicin resistant isolates). Two isolates did not test postitive for the bifunctional enzyme: strain JH1 (ST 40), for which the 3′5″ APH gene was previously characterized [56], and strain YI6-1, for which the mechanism was not identified. Aminoglycoside resistance via *aac6′-aph2″* first appeared in our collection in strain HH22 (ST6, CC2) in 1981, which was also the first identified β-lactamase-producing *E. faecalis* [95]. Aminoglycoside resistance was previously identified in HH22, in the first publication describing high-level gentamicin resistance in the U.S. [101]. Gentamicin resistance transferred by conjugation categorically with β-lactam resistance from HH22 [95], [101], signifying that resistance to both antibiotics was acquired simultaneously in this strain. In fact, all β-lactamase positive isolates in our collection are also resistant to high-levels of gentamicin, though a genetic link between these determinants was not investigated. The next occurrence of *aac6′-aph2″* in our set was in ST9 and ST106 (both CC9) in the mid-1980s, followed by ST64 (CC8) in 1986, ST11 (distant relative of CC9) and ST109 (CC2) in the early 1990s. The bifunctional gene was next found in ST28, a single locus variant of ST11, in the mid-1990s. By 2001/2002 the *aac6′-aph2″* had expanded to ST2 (CC2), ST4, ST103, and ST104; the gene also spread to ST16, however the date of this transfer is not known. The pervasive spread of this determinant among different lineages in the species hints that persistent use of aminoglycosides in the last several decades has permitted the expansion of this determinant between enterococcal isolates.

Erythromycin, a macrolide antibiotic, came into use in 1952. Within a year, resistant isolates of group D streptococci (enterococci) were identified [86]. Many genes have been found to contribute to macrolide resistance in enterococci, however resistance to erythromycin in *E. faecalis* is typically mediated by the *ermB* rRNA methylase gene [102]. Within our collection, all 40 erythromycin resistant isolates contained the *ermB* gene. The first occurrence of *ermB* in our group was in ST40 (CC40) and ST55 from the mid 1970s. By the mid 1980s, *ermB* had spread to ST6 (CC2), ST9 (CC9), and ST64 (CC8). By the early 1990s, *ermB* could be found in five additional sequence types, and by 2002 *ermB* had been transferred to an additional seven sequence types. In total, 17/51 sequence type lineages were found to contain the *ermB* gene for macrolide resistance. In addition, all vancomycin resistant strains in our study (either *vanA* or *vanB* mediated) harbored the *ermB* gene. The *vanA* gene was previously shown to be located on the same plasmid as *ermB* [103], though we could find no prior work describing linkage of *vanB* and *ermB*. Antibiotic resistance genes are commonly clustered together on mobile plasmids and phages, leading to the co-selection of resistance determinants with the use of a single class of antibiotic. Moreover, antibiotic treatment of *E. faecalis* infections often involves the use of multiple antibiotics to achieve synergistic killing effects [104]; thus the likelihood of a strain acquiring multiple antibiotic resistances at once is great.

The occurrence of PAI genes and acquired antibiotic resistances by clonal group reveals a pattern between the range of isolation dates and the average number of these genes observed: generally, the more PAI and antibiotic resistance genes found, the shorter the range of isolation dates within the clonal clusters. The five most common clonal clusters: CC21, CC9, CC2, CC8, and CC40, represented 58% of the 106 isolates examined by MLST in this study. CC2 isolates have the shortest range of isolation dates of any clonal group (1981–2003, 22+ years) and the greatest number of PAI and antibiotic resistance genes on average (7.5, ±1.8), while CC21 have the widest range of isolation (1930s–2006, 75+ years) and the least number of PAI and antibiotic resistance genes (1.4, ±1.2). CC9 strains have a range of 31+ years and 6.8 PAI/antibiotic genes (±1.5), CC8 have a range of 60 years and 5.8 (±3.5), and CC40 span 54+ years with 3.6 of these genes on average (±1.4). The non-ST64 isolates of CC8 alter the age range and genetic content of the group significantly, and if left out, change the range to 13 years and the average to 7.3 (±2.5). These patterns point to sudden clonal expansion of groups with greater virulence potential and mechanisms for resistance, and the persistence of clusters over time with few PAI genes and antibiotic resistances. Clusters with the most PAI genes sampled carried the most acquired antibiotic resistance traits (Fig. 3), suggesting that some lineages—particularly CC2—may be especially proficient at DNA exchange. Since the PAI is not physically linked to antibiotic resistance [10], this also indicates that there is independent selection for both antibiotic resistance and increased virulence. As has been observed in other species, the non-grouping/singleton isolates of our collection often contained fewer PAI and antibiotic resistance genes than isolates belonging to genetically similar clusters [105]–[107].

The frequency of occurrence of genes sampled from the pathogenicity island by lineage (51 STs) was: the *psaA* homolog 49% (25/51), *esp*, 47% (24/51), glycosyl hydrolase 41% (21/51), *cylB* 39% (20/51), *nuc1* 37% (19/51), and *gls24*-like 20% (10/51). 15 sequence types (29%) had none of the tested PAI genes. The majority of *gls24*-like positive isolates were found in CC2. Differences in the frequency of various segments within the island, even within a genetic lineage, suggests that there are modules within the island that can vary independently of the whole, as was proposed by other studies [10], [32]. The nuclease homolog (*nuc1*) was not found in any isolate from CC21 or in any strain isolated prior to 1950. Some older strains (isolated ≤1950) were positive for the accessory traits bile salt hydrolase, cytolysin, and gelatinase, as well as the PAI-associated genes *cylB, esp, hyd,* and *psaA.* Two PAI genes appear to have entered the species relatively recently: *nuc1*, which appeared by 1951, and *gls24*-like (EF0117), which appeared in isolates of our collection during the mid-1980s. This *gls24-*like gene appeared in our collection in other sequence types in the early 1990s in genetically distinct isolates: namely STs 11, 28, 36, and 64. These data suggest the *gls24-*like region of the pathogenicity island is a more recent acquisition to the species, however additional samples isolated prior to the 1980s would be needed to identify the lineage and date in which this portion was attained.

The PAI genes *esp, hyd, psaA,* and *cbh,* were found in isolates of our collection dating as far back as 1926. Cytolysin and gelatinase activity were present in an isolate dating from 1918 (SS-7) however, both traits were described in the 1898 report of enterococcal disease (then *Micrococcus zymogenes*) by McCallum and Hastings [108], indicating both entered the species before the 20^th^ century.

Because we have identified the regions of the pathogenicity island between the *esp* gene (EF0056) and the *psaA* homolog (EF0095) in isolates dating back to 1926, we propose that most of the elements of the known pathogenicity island have been present in this species for a minimum of 80 years, as have the auxiliary traits tested. Whether they occur as a single genetic element in these strains remains to be determined. As most *E. faecalis* do not cause infections, the high incidence of the auxiliary bile salt hydrolase and gelatinase genes in the population suggests these traits could have important roles in the commensal existence of many *E. faecalis* lineages. Gelatinase in particular, has been shown to affect the surface of the cell altering its behavior in biofilm formation, and increases the lethality of infections [109]–[111]. However, its ubiquity within the population appears to be due to a more central role in the commensal behavior of the species.

All strains isolated before the mid-1980s that were positive for cytolysin easily transmitted this activity to a non-cytolytic recipient strain by mating. Movement of the cytolysin operon to the pathogenicity island, as typified in strain MMH594 [10], appears to coincide with the emergence of the earliest ST6 lineage strains in hospital wards. Interestingly, strain MMH594 is the only isolate in CC2 of our collection that possesses an intact cytolysin operon and is phenotypically cytolytic. Strains V583 and V587 are known to have partial deletion of the cytolysin operon and IS element insertions, respectively [10]. It is possible that the other *cylB+* strains in CC2 carry cytolysin genes chromosomally and that these operons have been made inactive as V583 and V587 have, however we did not test this further. The selection forces for this variation, either *in vitro* or *in vivo*, remain unknown, but may represent the cyclic generation of unusually virulent lineages, their establishment within a niche, and then their gradual attenuation. Interestingly, integration of the cytolysin operon into the PAI coincided in time with the appearance of the *gls24*-like gene (EF0117) in the pathogenicity island. The appearance of both regions occurred in ST6 (CC2), providing further evidence that this lineage may be particular adept at acquiring exogenous genes through recombination. Support for this proposition is found in the observation that of the 14 variable trait genes specifically analyzed in all strains in this study (*nuc1, cylB, esp, hyd, psaA, gls24, blaZ, cat, vanA, vanB, ermB, aac6′aph2″, tetL, and tetM*), all of these genes occurred at the greatest rate in lineages harboring the CPS 2 capsule locus polymorphism (which includes ST6) (Fig 7). All of this data supports a model where entry of new traits into the species mainly occurs first through a CPS 2 strain (where they are found with the greatest frequency), then radiates outward through horizontal exchange. Alternatively, entry and exit of mobile elements through the species could occur randomly, with strains of the CPS 2 polymorphism simply having a greater tendency to fix these traits through recombination; and/or strains of the CPS 2 capsule polymorphism occur in sites where virulence traits and antibiotic resistances are of greater selective advantage, *i.e*., infected sites, and hence become fixed in these lineages.

We selected strains representing the 7 deepest nodes spanning the species dendrogram to identify those traits that are common to strains representing the maximum diversity within the *E. faecalis* species. This approach also allowed us to assess the extent to which all other known variable traits had penetrated into the species. It also allowed us to identify those genes that represent the core genome for *E. faecalis.* To the extent that variable gene content reflects niche selection, generation of a dendrogram of greatest relatedness in gene content, as revealed by comparative hybridization, would be predicted to cluster strains according to those that inhabit similar niches. This is in contrast to an MLST relatedness dendrogram, which measures genetic distance of strains. Clustering of isolates based on gene content by comparative hybridization analysis is also useful for identifying large scale insertions and deletions, which cannot be evaluated by MLST analysis. Large regions of the V583 genome were noted to be absent in other strains (represented as white in Fig. 5). Previously identified elements of presumptive extra-enterococcal origin make up the basis for most of these gaps. PHAGE02 (Fig. 5, region 4 [19]) appears to have entered the species very early in its history, is highly infectious, or includes an essential function, as all eight isolates we evaluated were positive for this element. We therefore operationally include the region previously identified as PHAGE02 as part of the core genome of *E. faecalis.* To our knowledge, this is the first phage associated with the core genome of a bacterial species. PHAGE02 has highest homology to the *Lactococcus lactis* bacteriophage Tuc2009 and the *Streptococcus thermophilus* bacteriophage Sfi11, however, based on the V583 genomic sequence, PHAGE02 appears to be less than half the size (15.3 kb) of either Tuc2009 or Sfi11 (39 and 39.8 kb, respectively) [112], [113]. The major structural genes of PHAGE02 do not appear to have deteriorated to pseudogenes, as often happens when loss of function of an element occurs, yet whether PHAGE02 represents a functional phage or is merely a remnant of a once-operative bacteriophage, is not clear. The advantage or selective pressure that has allowed this region to persist is not obvious and is the subject of ongoing work. In contrast, no pathogenicity island ORFs are represented in all eight isolates examined by comparative microarray hybridization. This would be consistent with a more limited role for a pathogenicity island for an organism that is mainly a commensal, or it may reflect a more recent acquisition to the species.

Because *E. faecalis* is capable of colonizing a wide range of hosts and environments, variants better suited for a particular niche would proliferate and fill that niche, leading to well defined genetic lineages of the species. Niche-specific phylogenetic lineages occur in other species, such as the plant pathogen *Pseudomonas syringae,* for which MLST analysis revealed clusters of isolates that were confined to particular plant hosts [114]. A particularly virulent and antibiotic resistant lineage of *E. faecalis* emerged in the mid 1980's that harbored an antiphagocytic capsule [44], a collection of auxiliary virulence genes encoded on a pathogenicity island [10], and multiple antibiotic resistances [19]. Interestingly, we found no SLVs or DLVs of this lineage (CC2) that do not possess multiple virulence traits and antibiotic resistances. The selective forces that led to the convergence of these traits, the mechanisms involved and the origins of this strain remain the subjects of ongoing study.

Additional strain references:

[115]–[156]

Additional table references:

[157]–[160]

## Supporting Information

Figure S1 Schematic of the pathogenicity island of E. faecalis strain MMH594. Dashed marks designate the approximate site on the island where gene products were assessed by PCR&Southern hybridization for comparative analysis of PAI components in reference strains. Each product is an amplification of the gene listed directly above and is represented in Fig. 2 by the corresponding letter designation.
(0.05 MB TIF)

Table S1 Supplemental Table 1–Primers used in this study
(0.04 MB XLS)

Table S2 Supplemental Table 2–Strain data chart
(0.19 MB XLS)
