# Supplementary figures and images for: Genetic Diversity among Enterococcus faecalis
Source: PLoS One. 2007 Jul 4;2(7):e582. doi: 10.1371/journal.pone.0000582 (PMC1899230; doi:10.1371/journal.pone.0000582)

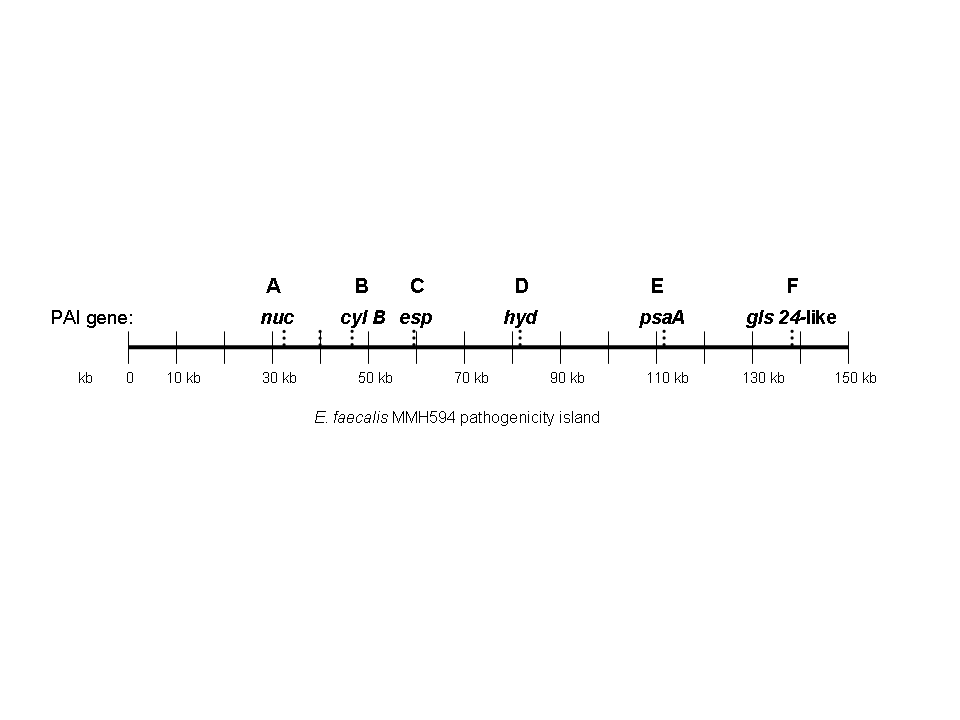

Supplement: Figure S1 — Schematic of the pathogenicity island of E. faecalis strain MMH594. Dashed marks designate the approximate site on the island where gene products were assessed by PCR&Southern hybridization for comparative analysis of PAI components in reference strains. Each product is an amplification of the gene listed directly above and is represented in Fig. 2 by the corresponding letter designation. (0.05 MB TIF) [file pone.0000582.s001.tif]
